# ATAC‐seq exposes differences in chromatin accessibility leading to distinct leaf shapes in mulberry

**DOI:** 10.1002/pld3.464

**Published:** 2022-12-15

**Authors:** Lei Wang, Yuming Feng, Jiangying Wang, Xin Jin, Qiaonan Zhang, Michael Ackah, Yuhua Wang, Dayong Xu, Weiguo Zhao

**Affiliations:** ^1^ School of Biology and Technology Jiangsu University of Science and Technology Zhenjiang China; ^2^ Leisure Agriculture Laboratory Lianyungang Academy of Agricultural Sciences Lianyungang China

**Keywords:** ATAC‐seq, biochemical, leaf shape, mulberry

## Abstract

Mulberry leaf shape is an important agronomic trait indicating yield, growth, development, and habitat variation. China was the earliest country in the world to grow mulberry for sericulture, and it is also one of the great contributions of the Chinese nation to human civilization. ATAC‐seq (Assay for Transposase Accessible Chromatin using sequencing) is a recently developed technique for genome‐wide analysis of chromatin accessibility. The samples used for ATAC sequencing in this study were divided into two groups of whole leaves (CK‐1 and CK‐2) and lobed leaves (HL‐1 and HL‐2), with two replicates in each group. The related motif analysis, differential expression motif screening, and functional annotation of mulberry leaf shape differences were performed by raw letter analysis to finally obtain the transcription factors (TFs) that lead to the production of heteromorphic leaves. These transcription factors are common in plants, especially the TCP family, shown to be associated with leaf development and growth in other woody plants and are a potential transcription factor responsible for leaf shape differences in mulberry. Dissecting the regulatory mechanisms of leaf shape of different forms of mulberry leaves by ATAC‐seq is an important way to protect mulberry germplasm resources and improve mulberry yield. It is conducive to cultivating mulberry varieties with high resistance to adversity, promoting the sustainable development of sericulture, and protecting and improving the ecological environment.

## 1 INTRODUCTION

Mulberry is a perennial heterogeneous, pollinated woody plant used in sericulture (Ma et al., 2019). *Morus alba* (scientific name: *Morus alba* Pendula) is a cultivated species of mulberry in the family Moraceae, which is distributed in tropical, subtropical, and temperate regions and has a wide distribution in China. It can also form mixed forests with other trees, which increases species diversity and ecological stability. Mulberry has a large biomass and carbon storage capacity and has various ecological functions such as soil and water conservation, wind and sand control, and air purification. As a roadside landscape tree, it has the function of beautifying the environment (Jokar et al., 2016).

Mulberry leaves are composed of a petiole, leaf blade, and stipules, and are the main assimilation organs and harvests of mulberry trees. In nature, the same plant can display both complete and lobed leaves, and because both are derived from the same genetic background, the differences in their phenotypes are regulated by epigenetics (Itabashi et al., 2018; Zhao & Zhou, 2012). Leaf morphological differences have an important role in variety identification, sericulture production, and natural disease control. Leaf shape is an important agronomic trait in mulberry because the size and shape of mulberry leaves are closely related to economic yield. The leaf shape also reflects differences in energy metabolism, variations in tree habitats, and genetic variation (Cao et al., 2019; Ramesha et al., 2020; Samami et al., 2019). The study on the growth and morphogenesis of mulberry leaves and the mechanisms underlying variation in leaf shape can provide strong support for future mulberry as a model woody plant. The pendant mulberry leaf types, including *M. alba* Pendula can vary between unlobed and up to 6–7‐lobed. Therefore, *M. alba* Pendula provides a most suitable research material to study epigenetic mechanisms of leaf shape regulation in mulberry.

In recent years, the formation of leaf shape has been studied extensively. Detailed morphological observations of leaf development from meristem cell initials has led to the identification of growth centers and the development of predictive models of polarized growth along proximal‐distal, medial‐lateral, and adaxial‐abaxial axes in simple and complex leaf structures (Diaz, 2017). Some of the genes regulating growth along the different axes and their interplay have been reported (Itakura & Hosoi, 2018). However, the regulatory networks underlying the formation of different leaf shapes from the same genome, such as in mulberry, has received little attention to date. Therefore, the aim of this work is to identify, study, and utilize key TFs governing the formation of different mulberry leaf morphologies. A greater understanding of the regulatory mechanisms underlying the production of different mulberry leaf morphologies is an important way to conserve mulberry germplasm resources and improve mulberry yield. It is also conducive to breeding mulberry varieties with high resistance to adversity, promoting sustainable development of sericulture and protecting and improving the environment.

ATAC‐seq is a recently developed technique for genome‐wide analysis of chromatin accessibility. Compared with earlier ChIP‐seq or DNase‐seq methods for detecting chromatin accessibility, ATAC‐seq is faster and easier to perform, does not require cross‐linking, has a higher signal‐to‐noise ratio, and can be performed on a smaller number of cells. ATAC‐seq takes into account transposase‐digested DNA fragments that contain other nucleosome localization information (Lu et al., 2021). ATAC‐seq seq is widely used to measure chromatin accessibility and to identify open chromatin regions (OCRs). OCRs usually indicate active regulatory elements in the genome and is directly related to gene regulatory networks. Identification of differentially accessible regions (DARs) between different biological conditions is essential to determine the differential activity of regulatory elements. Differential analysis of ATAC‐seq data has many similarities to differential expression analysis of RNA‐seq data. However, the distribution of ATAC‐seq signal intensity differs from that of RNA‐seq data, and DAR identification requires higher sensitivity. Many different tools can be used to perform differential analysis of ATAC‐seq data (Gontarz et al., 2020).

In our study, we have analyzed the motifs associated with leaf shape differences in mulberry through chromatin accessibility assay of and identified the associated TFs through differential expression motif screening, functional enrichment, and annotation, thus providing new information of molecular mechanisms involved in the generation of heteromorphic leaves in mulberry.

## 2 MATERIALS AND METHODS

### 2.1 Plant materials

*M. alba* Pendula were obtained from the Nursery of the National Mulberry Germplasm at Jiangsu University of Science and Technology, Zhenjiang, China. Young whole and lobed leaves were sampled in early April. Furthermore, fresh, young, and actively growing tissues were removed from mulberry trees. Dust or soil from the surface of the material were rinsed with sterile water and blot it up. Large tissues were cut into small pieces or thin slices on ice and then placed into 2 ml screw‐cap lyophilization tubes of >400 mg each, labeled with the sample name; quickly submerged in liquid Nitrogen for at least 1 h of snap‐freezing and then stored in a −80°C refrigerator for subsequent experiments.

### 2.2 Flow chart of experiments

Nuclei suspensions were incubated in a Transposition Mix that includes a Transposase. The Transposase enters the nuclei and preferentially fragments the DNA in open regions of the chromatin. Simultaneously, adapter sequences are added to the ends of the DNA fragments. The transposition reaction was incubated at 37°C for 30 min. Immediately following transposition, the products were purified using a QIAGEN mini‐elute kit, amplified as described in (Buenrostro et al., 2013), and sequenced using Illumina HiSeqTM 4000 by Gene Denovo Biotechnology Co. (Guangzhou, China).

### 2.3 Clean reads filtering

Reads obtained from the sequencing machines were filtered to remove adapter sequences, reads containing more than 10% of unknown nucleotides (N) and low quality reads containing more than 50% of low quality (Q value ≤ 20) bases.

### 2.4 Reads alignment

Bowtie2 (Langmead & Salzberg, 2012) (version 2.2.8) with the parameters”–X 2000″ was used to align the clean reads from each sample against the reference genome, and reads aligned to the mitochondria or chloroplasts were filtered out. For all data files duplicates were removed using Picard.

### 2.5 Peak scanning

All reads aligning to the + strand were offset by +4 bps, and all reads aligning to the – strand were offset −5 bps. The shifted, concordantly aligned paired mates were used for peak calling by MACS (version 2.1.2) (Zhang et al., 2008) with the parameters “‐‐nomodel ‐‐shift ‐100 ‐‐extsize 200 ‐B ‐q 0.05.” Dynamic Poisson Distribution was used to calculated The *p*‐value of the specific region based on the unique mapped reads. The region was defined as a peak (accessible DNA region) when the *q*‐value <.05.

### 2.6 Peak‐related genes annotation

According to the genomic location information and gene annotation information of the peak, peak‐related genes were confirmed using ChIPseeker (Yu et al., 2015) (version v1.16.1). The position of the peak on the gene or inter‐genic regions (such as promoter, 5′UTR, 3′UTR, exon, intron, downstream and intergenic) was recorded.

### 2.7 Peak‐related genes GO enrichment analysis

Gene ontology (GO) is an international standardized gene functional classification system which offers a dynamic‐updated controlled vocabulary and a strictly defined concept to comprehensively describe properties of genes and their products in any organism. GO has three ontologies: molecular function, cellular component and biological process. The basic unit of GO is the GO term. Each GO term belongs to a type of ontology. GO enrichment analysis provides all GO terms that significantly enriched in peak‐related genes comparing with the genome background, and filter the peak‐related genes that correspond to biological functions.

All peak‐related genes were mapped to GO terms in the Gene Ontology database (http://www.geneontology.org/) and gene numbers were calculated for every term. Significantly enriched GO terms in peak‐related genes comparing with the genome background were defined by the hypergeometric test. The formula for *p*‐value calculation was P=1−∑i=0m−1MiN−Mn−iNn, where *N* is the number of all GO annotated genes; *n* is the number of peak‐related genes in *N*; *M* is the number of all genes that are annotated to the certain GO terms; *m* is the number of peak‐related genes in *M*. The calculated *p*‐value was FDR corrected, taking FDR ≤ .05 as the threshold. GO terms meeting this condition were defined as significantly enriched GO terms in peak‐related genes.

### 2.8 Peak‐related genes pathway enrichment analysis

KEGG pathway (Kanehisa et al., 2008) enrichment analysis was used to identify significantly enriched metabolic pathways or signal transduction pathways in peak‐related genes relative to the whole genome background. The calculating formula is the same as that in GO analysis. Here, *N* is the number of all transcripts that with KEGG annotation, *n* is the number of peak‐related genes in *N*, *M* is the number of all transcripts annotated to specific pathways, and *m* is number of peak‐related genes in M. The calculated *p*‐value was FDR corrected, taking FDR ≤ .05 as a threshold. Pathways meeting this condition were defined as significantly enriched.

### 2.9 Irreproducible discovery rate (IDR)

To measure consistency between biological replicates within an experiment, Irreproducible discovery rate (IDR) (Li et al., 2011) software (version 2.2) was used with parameters “‐‐input‐file‐type narrowPeak –plot.” IDR calculations using scripts provided by the ENCODE project (https://www.encodeproject.org/software/idr/) were performed on all pairs of replicates using an oracle peak list called from merged replicates for each condition. Peaks showing an IDR value of .05 or less were considered relevant.

### 2.10 Motif analysis

The MEME suit (http://meme-suite.org/) was used to detect motifs in peak sequences. We used MEME‐ChIP to scan motifs with high reliability through peak regions, and nd used MEME‐AME to confirm the existences of any specific known motifs.

### 2.11 Differential analysis of multi‐samples

The DiffBind (Stark & Brown, 2011) software (version 2.8.0) was used to analyze peak differences across the two groups and significant differential peaks with an FDR of <.05 were selected. Using the same method, genes associated with the different peaks were annotated, and enrichment analysis of GO functions and KEGG pathways were identified.

#### 2.11.1 Transcription factors validation (TFs) by qRT‐PCR

Total RNA was extracted from fresh leaf samples using the RNAiso Plus reagent (Takara, China), according to the manufacturer's protocol. First, the concentration and purity of total RNA were checked on a Nanodrop spectrophotometer. Then, the extracted total RNA was reverse transcribed using PrimeScript™ RT reagent Kit (Takara, China), following the manufacturer's instructions, to obtain the cDNAs. Three‐step qPCR was performed using the FastStart Universal SYBR Green Master Mix kit (Roche, USA) in ABI Quant Studio 6 Flex System (Applied Biosystems, USA), according to the kit instructions. The qPCR reaction mixture comprised 10 μl FastStart Universal® SYBR Green Master, 1 μl forward primer (10 μM), 1 μl reverse primer (10 μM), 2 μl cDNA, and the addition of ddH_2_O to make a final volume of 20 μl. Amplification was achieved as follows; PCR cycling conditions: 95°C denaturation, 1 min, 35 cycles: denaturation at 95°C, 20 s; annealing at 60°C, 20 s; extension at 72°C, 30 s.

## 3 RESULTS

### 3.1 Experimental library construction process and data quality control

Cell samples were obtained and the nuclei were extracted, Tn5 transposase was added to the nuclei suspension for transposition reaction, and the DNA fragments were purified after the reaction was completed. The amplification products were then used for PCR amplification and purification to construct sequencing libraries. After the libraries were constructed, the libraries were tested for quality. Libraries that passed the quality check were used for up‐sequencing to obtain sequence information of the open chromatin region to be sequenced. Data filtering was performed on the downstream data to remove low‐quality data. The filtered data was then compared with the reference genome and after confirming the quality of the comparison, the sequences at the unique position on the comparison were extracted and processed for information analysis.

**FIGURE 1:**
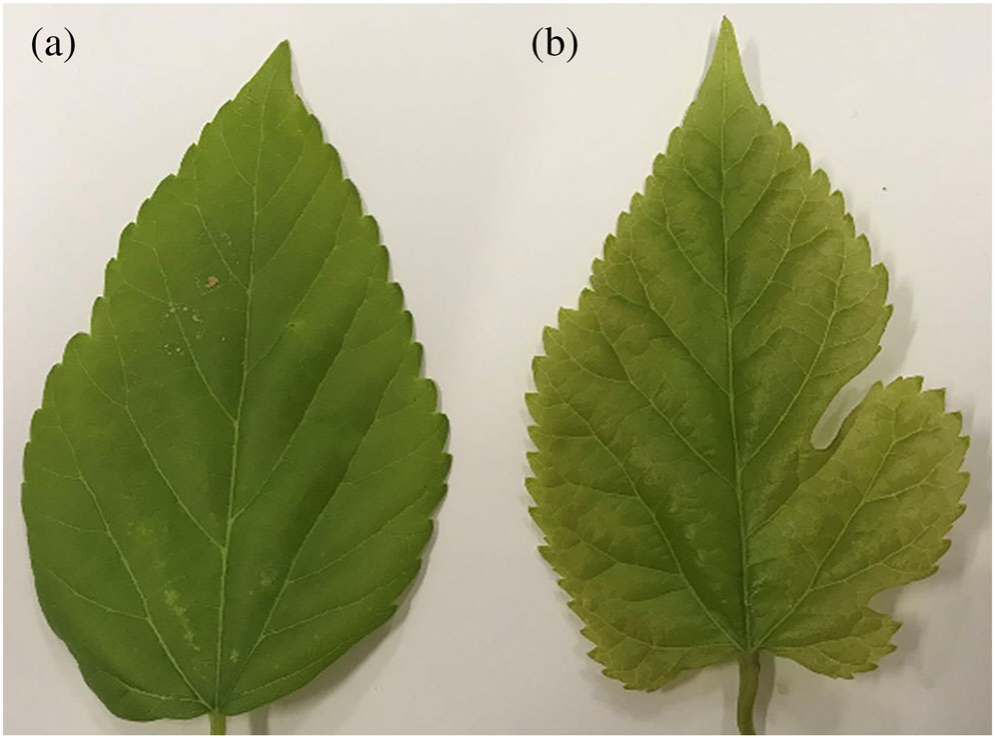
Mulberry leaf shape. (a) Whole leaf; (b) lobed leaf

**FIGURE 2:**
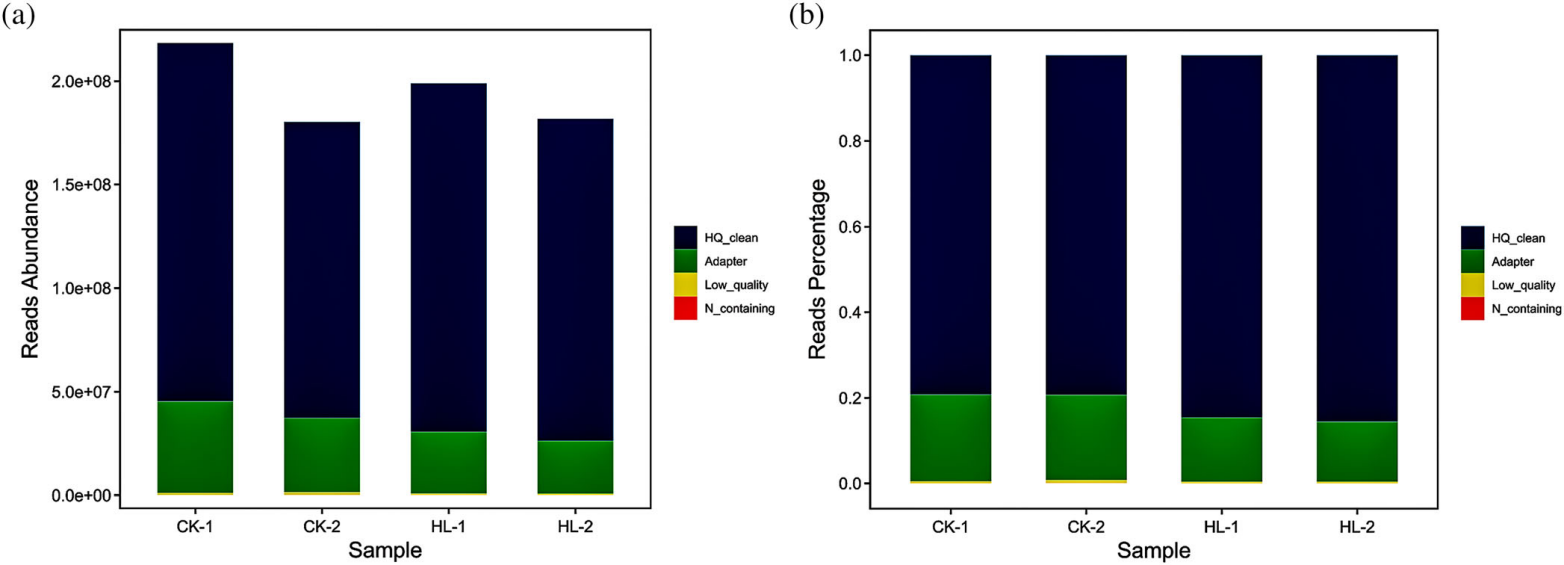
Data quality control. (a) Sample filtering analysis frequency graph; (b) sample filtering analysis proportion graph

The samples used for ATAC sequencing in this study were divided into two groups of whole leaves (CK‐1 and CK‐2) (Figure 1a) and lobed leaves (HL‐1 and HL‐2) (Figure 1b). The Clean reads obtained from the lower machine data after processing CK‐1, CK‐2, HL‐1, and HL‐2 were 217924734, 179669554, 198654016, and 181546126, respectively, with the percentage of low quality reads ranging from .17% to .34% (Table 1). Frequency and proportion plots of reads indicate that HQ_Clean_Reads_Num and Adapter account for a significant proportion (Figure 2). From the distribution of bases before and after data filtering, it can be seen that the quality of filtered data is higher (Figure S1).

**TABLE 1:** Reads filtered information statistics

Sample	Clean_Reads_Num	HQ_Clean_Reads_Num (%)	Read_length	Adapter (%)	Low_quality (%)	PolyA (%)	*N* (%)
CK‐1	217924734	173083490 (79.42%)	150 + 150	44368018 (20.36%)	946452 (.22%)	0 (.0%)	0 (.0%)
CK‐2	179669554	143046708 (79.62%)	150 + 150	35964666 (2.02%)	1236016 (.34%)	0 (.0%)	80344 (.02%)
HL‐1	198654016	168445512 (84.79%)	150 + 150	29878974 (15.04%)	659060 (.17%)	0 (.0%)	0 (.0%)
HL‐2	181546126	155636996 (85.73%)	150 + 150	25594048 (14.1%)	630164 (.17%)	0 (.0%)	0 (.0%)

### 3.2 Comparison analysis

**FIGURE 3:**
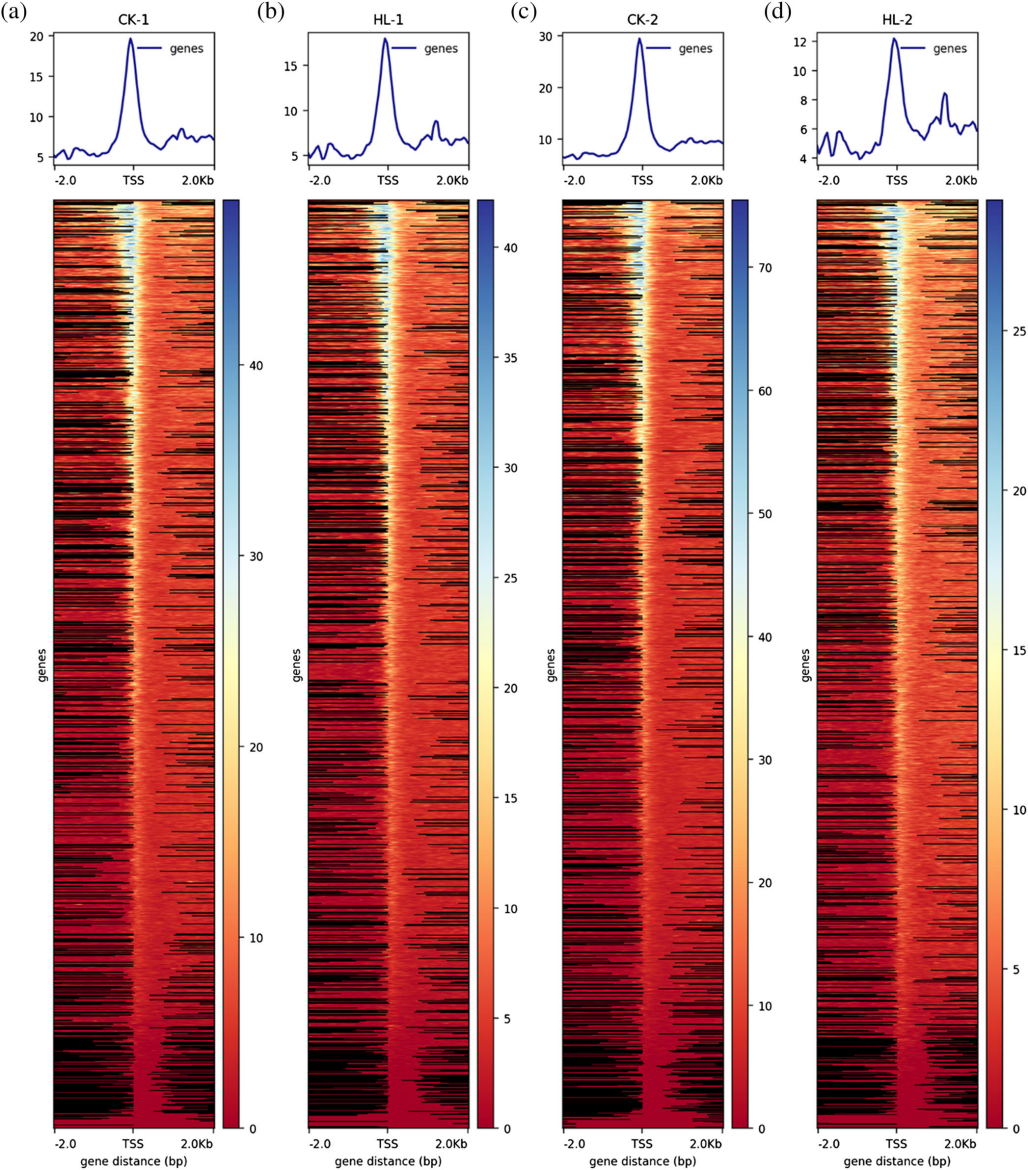
Distribution of reads relative to TSS position and signal. (a) Distribution of CK‐1 reads relative to TSS position and signal; (b) distribution of HL‐1 reads relative to TSS position and signal; (c) distribution of CK‐2 reads relative to TSS position and signal; (d) distribution of HL‐2 reads relative to TSS position and signal distribution

**FIGURE 4:**
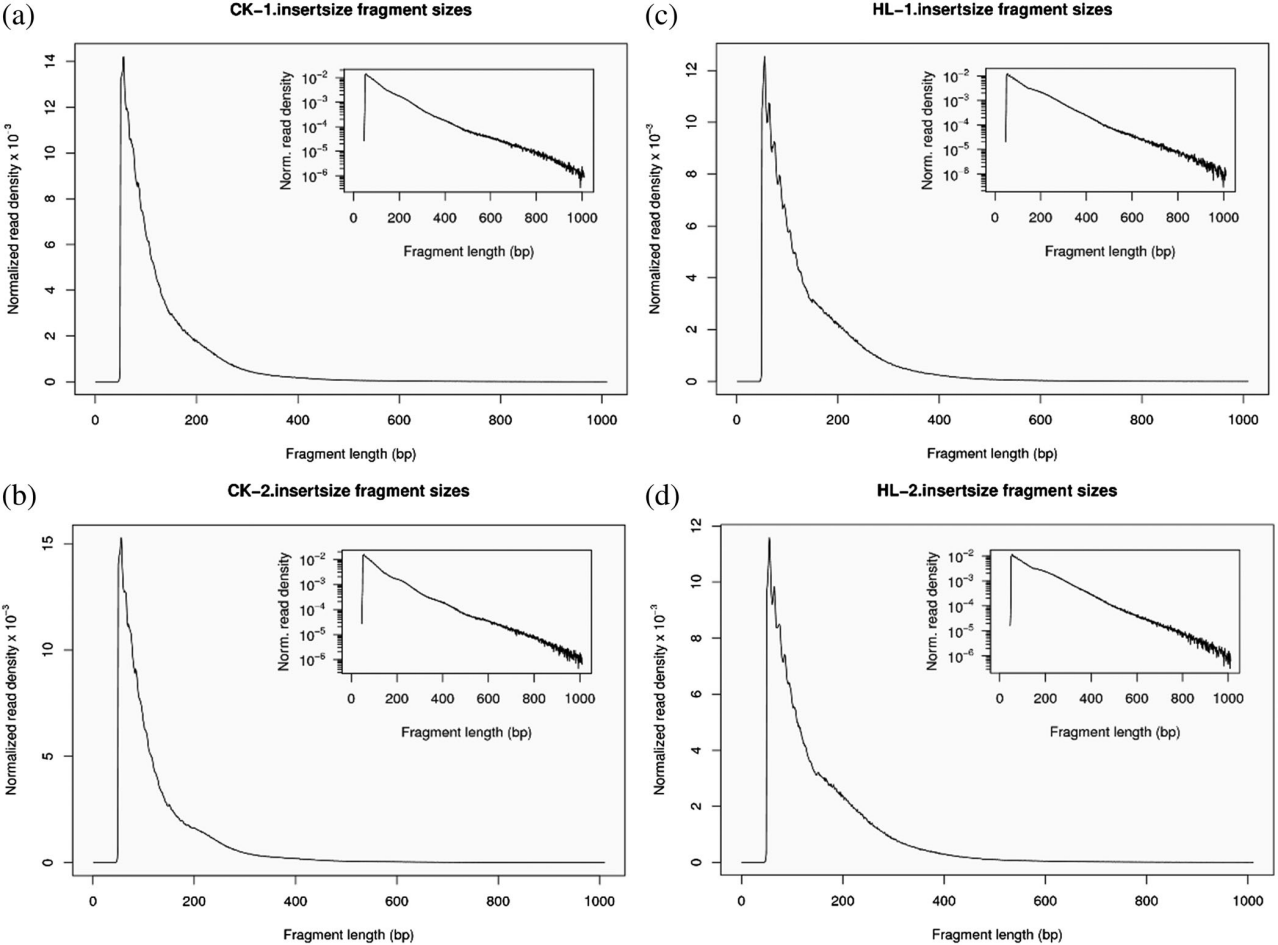
Distribution of insertion fragments. (a) Distribution of CK‐1 insertion fragments; (b) distribution of CK‐2 insertion fragments; (c) distribution of HL‐1 insertion fragments; (d) distribution of HL‐2 insertion fragments

The data were matched to the reference genome using the matching software Bowtie2, and reads that matched to mitochondria or chloroplasts were filtered out (Table S1). Reads matching to a unique position on the genome (unique matched reads) were used for the subsequent information analysis (Table 2). The unique matched sequences obtained after alignment were analyzed for their coverage distribution on the reference genome, and the depth information of genomic loci was counted to obtain the statistical results of sequencing depth on the genome (Figure S2). All reads in the 2 kb region upstream and downstream of the transcription start site (TSS) were counted using deepTools (Ramírez et al., 2016) software with a window of size 50 bp, and the average depth of reads within each window was calculated. The distribution of reads relative to the transcriptional start site (TSS) was plotted in a fold line and heat map (Figure 3). The densities of uniquely matched, de‐duplicated reads were compared with the individual chromosomes (divided into positive and negative strands) on the genome for statistical purposes (Figure S3). Because Tn5 transposase preferentially attacks the chromatin open region, in general, most of the DNA fragments produced are short and contain none or only one nucleosome, whereas there are also long fragments containing multiple nucleosomes, which will show distinct fragment distribution characteristics in terms of content distribution. The insertion fragment distribution of each sample was mapped using ATACseqQC (Ou et al., 2018) (Figure 4).

**TABLE 2:** Statistical results of each sample compared to the reference genome

Sample_ID	Total_Reads	UnMapped_Reads	Mapped_Reads	Multi_Mapped_Reads	Unique_Mapped_Reads	Duplicate_Reads
CK‐1	173083490	42058971‐24.30%	131024519‐75.70%	81154630‐46.89%	49869889‐28.81%	94977008‐72.49%
CK‐2	143046708	38573646‐26.97%	104473062‐73.03%	58335266‐40.78%	46137796‐32.25%	63862448‐61.13%
HL‐1	168445512	41443871‐24.60%	127001641‐75.40%	79120355‐46.97%	47881286‐28.43%	92423246‐72.77%
HL‐2	155636996	38035814‐24.44%	117601182‐75.56%	74137809‐47.64%	43463373‐27.93%	85131006‐72.39%

### 3.3 Single sample peek detection and statistics

**FIGURE 5:**
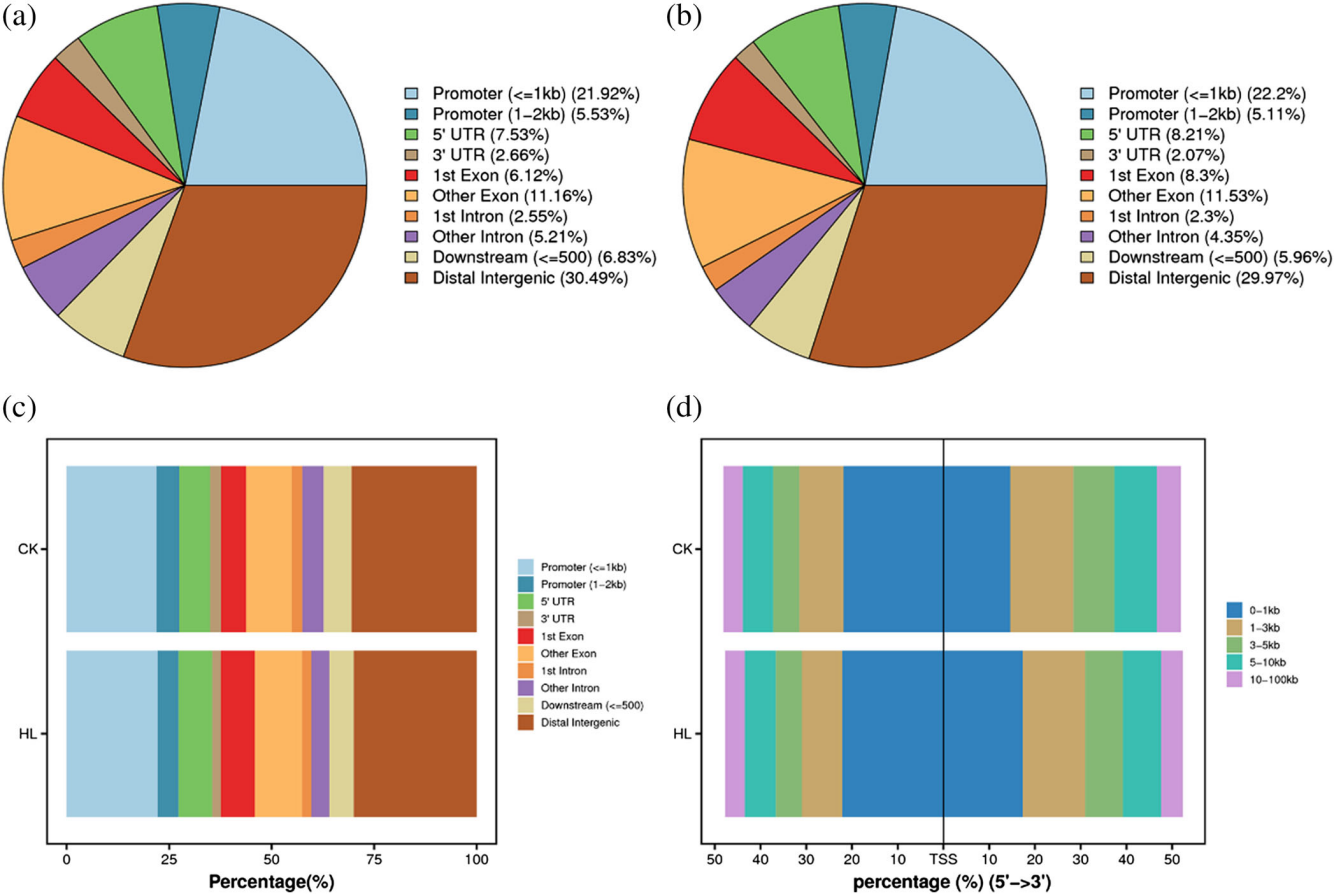
Distribution of shared peaks in functional elements of genes within the group. (a, b) Pie chart of distribution of shared peaks in functional elements of genes; (c) Proportion of distribution of shared peaks in functional elements of genes; (d) proportion of distribution of shared peaks relative to TSS distance

Sequencing analysis of DNA products after Tn5 transposase digestion was performed to find open chromatin regions from the whole genome. Because Tn5 transposase brings about an adaptor shift of four bases in the positive strand and five bases in the negative strand, the position of the reader needs to be corrected for displacement by shifting the reads position of the paired to the positive strand by +4 and the paired to the negative strand by −5. A genome‐wide peak (open chromatin enriched region) scan was performed using MACS2 analysis software (peak calling) with a threshold *q*‐value < .05. The peak sequences were then analyzed to identify their associated genes (Table 3). The distribution of peaks was counted (Table S2) and their genomic distribution classified as either promoter (within 2 k upstream of the gene), 5′UTR, 3′UTR, Exon, intron, downstream (within 500 bp downstream of the gene) or distal intergenic (beyond 2 k upstream of the gene or beyond 500 bp downstream) (Table S3), and determine the correspondence between the peak and each functional region (Figure 5).

**TABLE 3:** Number of peak statistics by sample

SampleId	PeakNumber	TotalLength	AverageLength	TotalPileup	AveragePileup	GenomeRatio	FRiP
CK‐1	31612	12689327	401	1163575	36	4.29%	65.81%
CK‐2	39097	16696817	427	1636508	41	5.65%	64.67%
HL‐1	29915	11876626	397	1002739	33	4.02%	69.85%
HL‐2	28070	10729635	382	773489	27	3.63%	68.87%

Where the location of peaks was classified as distal intergenic, their regulatory relationship with the nearest neighboring gene could not be established with any confidence. Therefore, peaks categorized as distal intergenic were eliminated from further consideration. The remaining peak‐related genes were used in GO and KO enrichment analyses (Figures S4 and S5).

### 3.4 Analysis of TFs associated with intra‐group shared peaks

**FIGURE 6:**
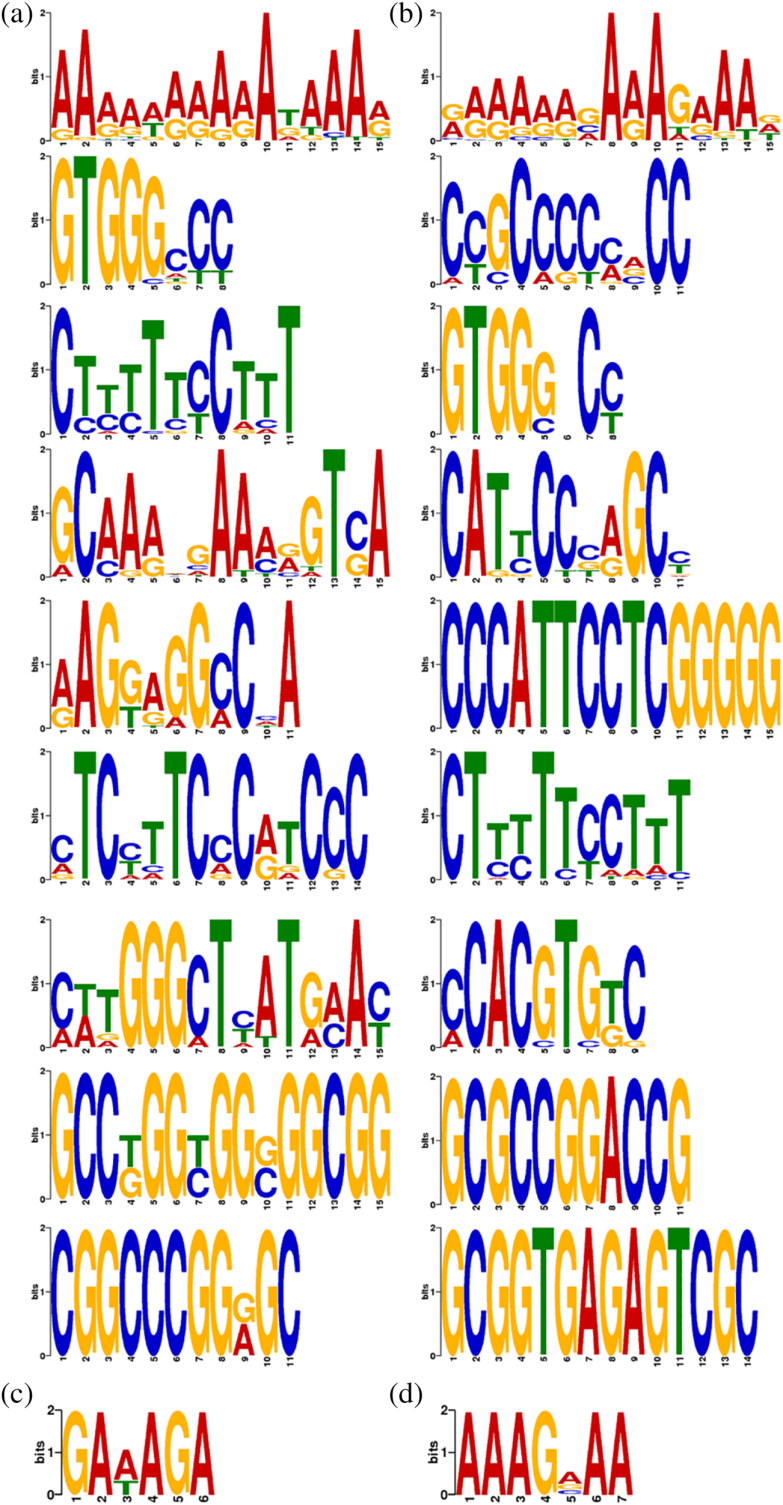
MEME significant motif sequence map

TFs are proteins that bind specific DNA regulatory motifs of genes to regulate their gene transcription. TF‐motif analysis was performed using MEME‐chip motif discovery of the MEME Suite (http://meme-suite.org/). The analysis included an enrichment analysis of known TF (present in the database) and predicted TF motifs. The top 400 peaks of the enrichment multiplicity were taken by default (Figure 6). Different colors represent different base types, and the height of the letter represents the conservativeness of the base (the higher the letter, the higher its frequency across the locus, the more conserved it is).

**FIGURE 7:**
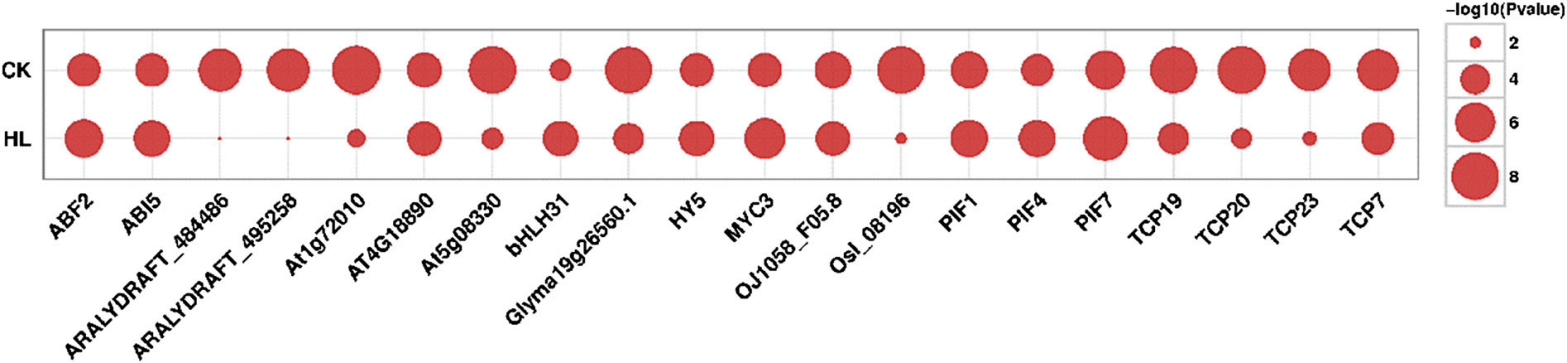
Motif enrichment bubble diagram

**FIGURE 8:**
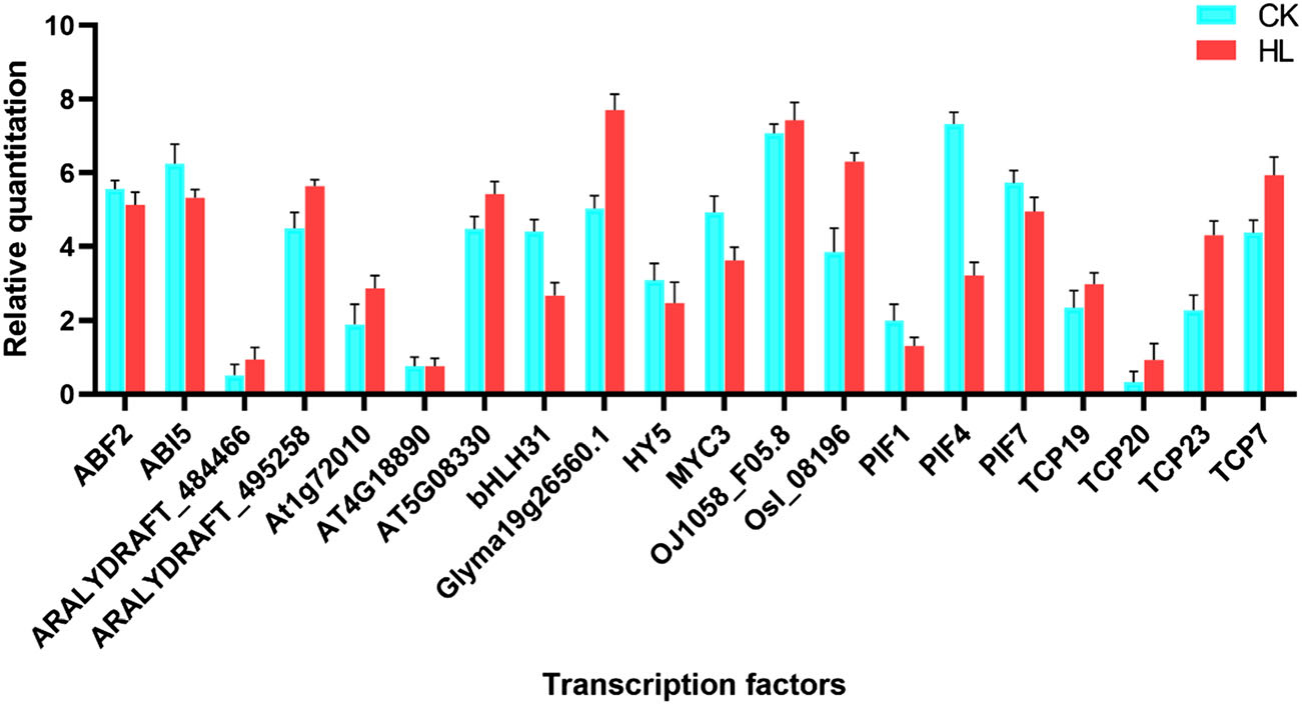
Transcription factors validation (TFs) by qRT‐PCR. CK: whole leaf; HL: lobed leaf.

Currently, a large number of DNA motifs recuiting TFs have been reported in transcription factor databases (e.g., JASPAR database). Enrichment analyses of these motifs helps us to quickly target the corresponding key TFs, which provides clues for further data parsing and downstream molecular experiments. Here, we have used Analysis of Motif Enrichment (AME) of the MEME Suite to perform enrichment analysis of TF motifs (Table S4). AME uses the JASPAR CORE non‐redundant database as a control for input sequence enrichment. Motifs with a significant *p*‐value were considered to be an enriched TF motif (Figure 7). The top 20 enriched transcription factors were ABF2, ABI5, ARALYDRAFT_484466, ARALYDRAFT_495258, At1g72010, AT4G18890, AT5G08330, bHLH31, Glyma19g2656.1, HY5, MYC3, OJ1058_F05.8, OsI_08196, PIF1, PIF4, PIF7, TCP19, TCP20, TCP23, and TCP7. These enriched transcription factors were verified by qPCR (Table S5), and the experimental results showed consistent trends between the two experimental methods, ATAC‐seq and qPCR (Figure 8).

### 3.5 Merging of peaks across treatment groups and multisample clustering

**FIGURE 9:**
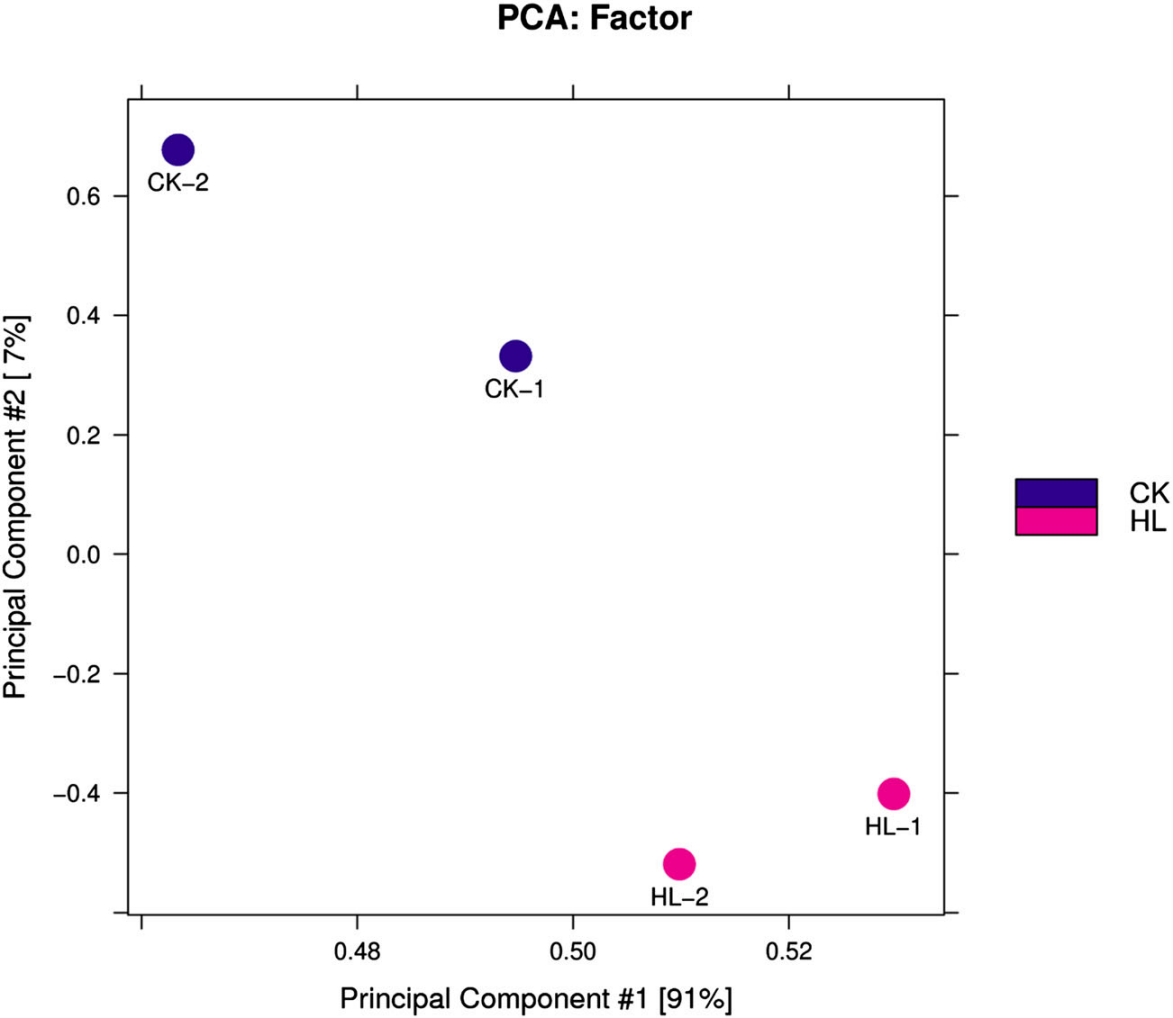
Sample principal component analysis

**FIGURE 10:**
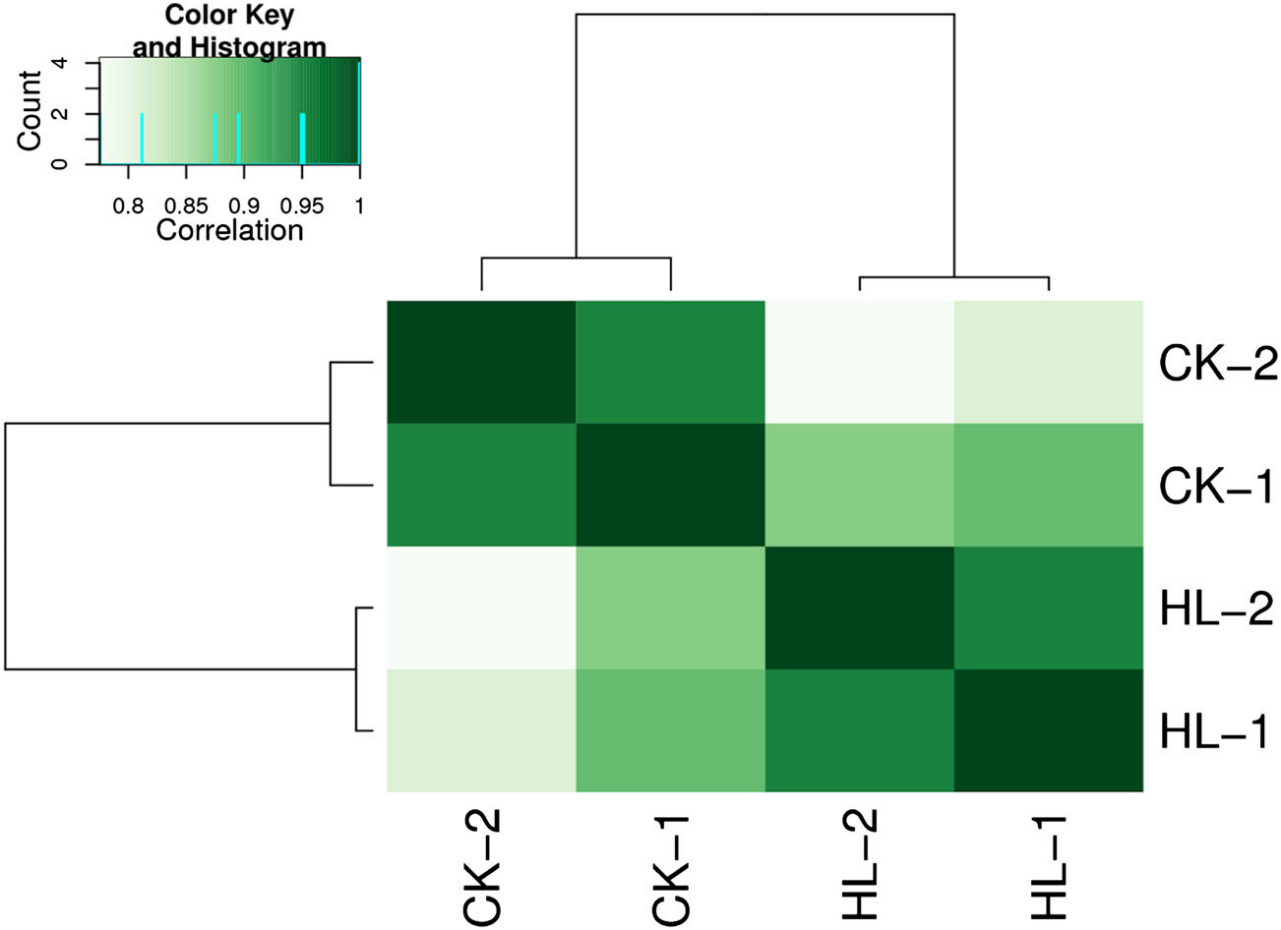
Sample correlation heat map

DiffBind software was utilized to perform the merging of peaks from each treatment group and to calculate the abundance of each peak in each sample. To facilitate inter‐treatment comparisons, principal component analysis (PCA) was used to reduce, the high dimensional information contained in the samples (thousands of peaks enriched with information) into a composite indicator of several dimensions (principal components), while ensuring that as much information as possible was retained in the original data (Figure 9). The Pearson correlation coefficients calculated between every two samples are presented visually in the form of a heat map (Figure 10).

### 3.6 Analysis of peaks and their associated genes shared or unique in simple and lobed leaves

**FIGURE 11:**
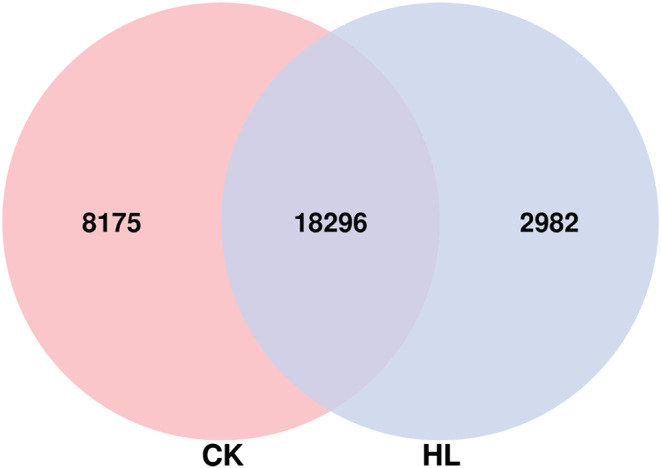
CK versus HL intergroup peak Venn diagram

A Venn diagram describes the common and unique peaks present in simple and lobed leaves (Figure 11). In particular, the peaks specific to each group were associated with the biological regulation specific to the corresponding group. For the peaks that are common or unique among the comparison groups, we further analyze the peak‐associated genes. The nearest neighboring gene of each peak were initially considered as the peak‐related gene. Considering that when peaks are far away from genes, it is difficult to determine whether there is a regulatory relationship between peaks and genes. Therefore, the peaks classified as distal intergenic were not considered further. The remaining peak‐related genes were subsequently subjected to GO (Figure S8) and KO enrichment analyses (Figure S9).

### 3.7 Analysis of TF motifs exclusively present in simple or lobed leaves

For peaks specific to a particular treatment group, TF motif analysis was performed use using the MEME Suite to predict conserved motifs within the peak region (400 peaks were taken by default). MEME‐chip was used to detect new motifs, and the AME package used to detect known motifs. Longer (8–15 bp) and shorter motifs (3–8 bp) were predicted from scratch using MEME software (Figure S10 A&B) and DREME software (Figure S10C,D), respectively. For the enrichment analysis of known TFs‐motifs, the number of grouped TF motifs were 35 and 77 (Table S6). Grouped enrichment statistics were performed (Table S7) and grouped motif enrichment bubble plots were obtained (Figure S11).

### 3.8 Quantitative analyses of the differential availability of peaks and associated genes between simple and lobed leaves

The qualitative analysis of peak and associated genes presented above indicated some TF binding sites that were differentially employed in simple and lobed leaves. However, quantitative differences in the availability of TF binding sites can also reveal potential differential regulations of genes between these two morphologies.

**FIGURE 12:**
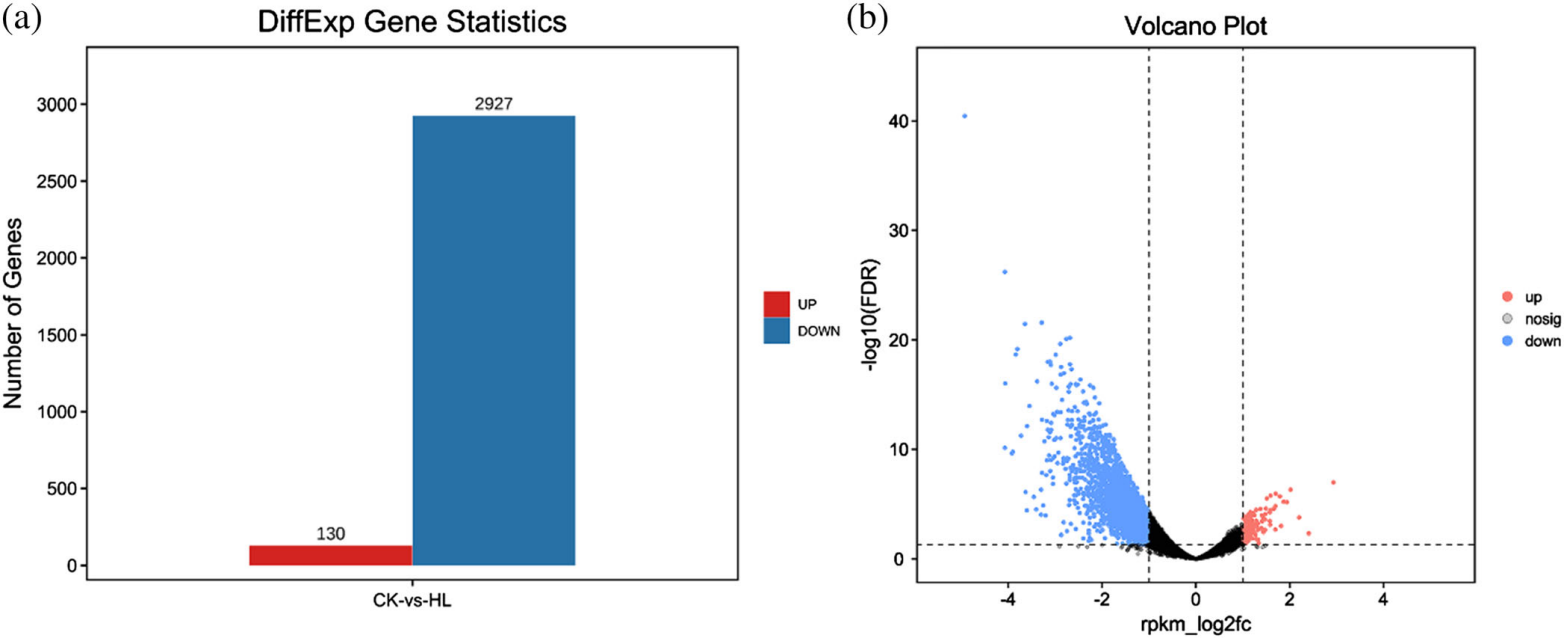
(a) Difference peak statistics plot; (b) CK versus HL difference comparison volcano plot. Up: difference up‐regulated peak; down: difference down‐regulated peak

**FIGURE 13:**
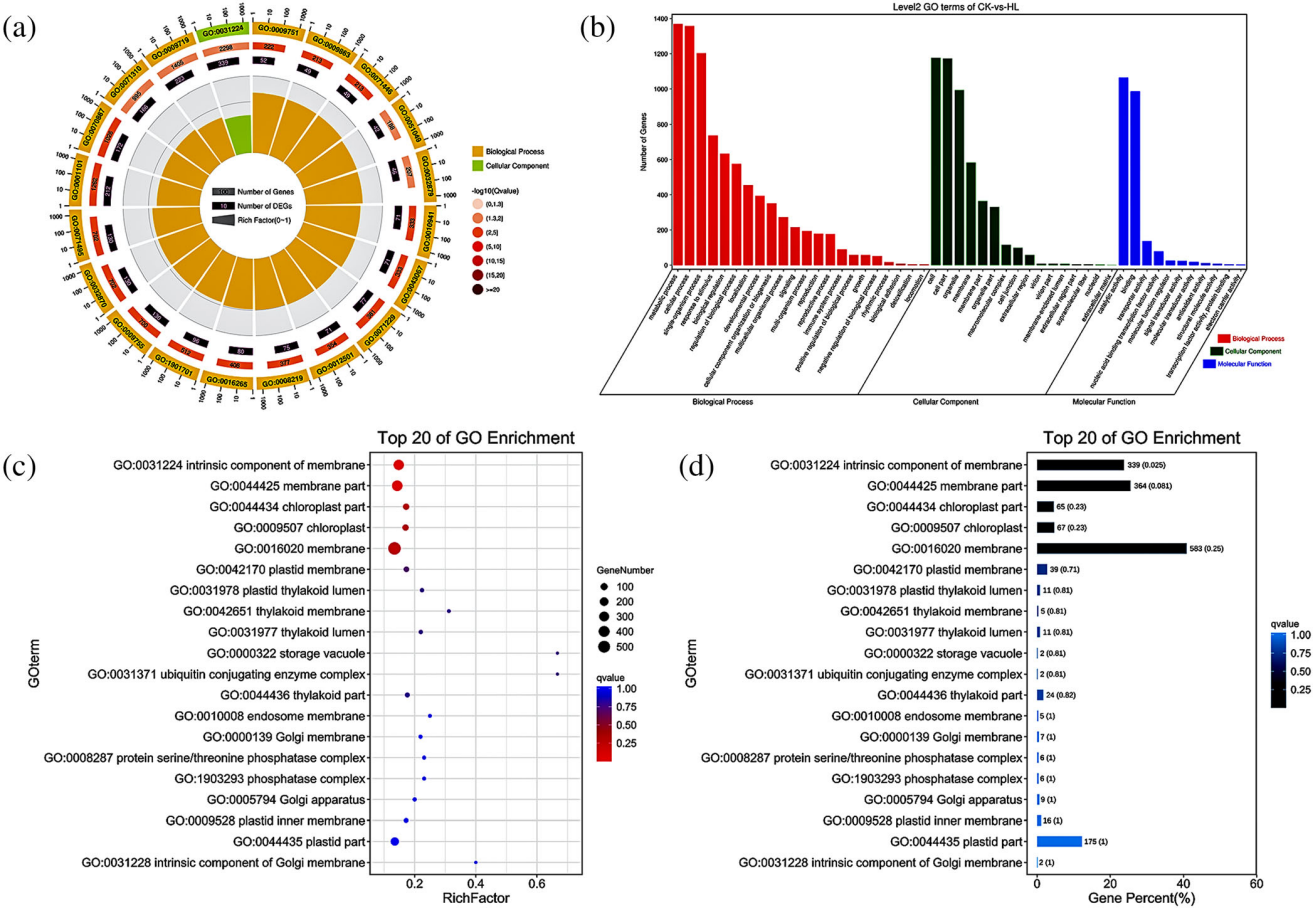
GO enrichment analysis. (a) GO enrichment circle graph. First circle: enrichment of the top 20 GO terms; outside the circle is the coordinate scale of gene numbers. Different colors represent different ontologies. Second circle: the number of GO terms in background genes and the Q value. Bar lengths reflect the number of genes and their Q values are mapped to red intensity, where smaller Q values are more intensely red. Third circle: the number of genes differing in the GO term. Fourth circle: the RichFactor value of each GO term (the number of differences in the GO term divided by the number of all). In the background grid, each cell represents a RichFactor unit of .1). (b) GO enrichment classification bar graph; (c) GO enrichment bubble graph, GO enrichment bubble graph of the top 20 GO terms with the smallest Q values. The vertical coordinate is GO term, the horizontal coordinate is the enrichment factor (the number of variances in that GO term divided by all the numbers), and the bubble size indicates how many and the darker the color the smaller the Q value). (d) GO enrichment bar graph (constructed using the first 20 GO term with the smallest Q values): The vertical coordinate is the GO term, and the horizontal coordinate is the number of that GO term as a percentage of the number of all variances. The darker the color, the smaller the Q value, and the values depicted on the bars are the number of GO term and the Q value. Different colors represent different A classes. Second circle: the number of pathways in the background genes and the Q value. The more genes the longer the bar, the smaller the Q value the redder the color. Third circle: the number of genes differing in the pathway. Fourth circle: the RichFactor value of each pathway (the number of differences in the pathway divided by the number of all), background grid lines, each cell represents .1 RichFactor unit). The vertical coordinate is the pathway, the horizontal coordinate is the enrichment factor (the number of differences in the pathway divided by the number of all), the size indicates the number of how much, and the more red the color the smaller the Q value. The darker the color the smaller the Q value, and the value on the bar is the number of that pathway and the Q value.

**FIGURE 14:**
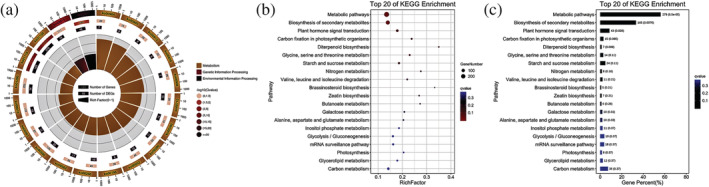
KO enrichment analysis. (a) KO enrichment circle; (b) KO enrichment bubble; (c) KO enrichment bar graph

We therefore used DiffBind software to identify the differential availability of peaks between simple and lobed leaf samples, where peak motifs with differences in abundance of log2 values ≥1 and a FDR ≤.05 were selected and their peak‐associated genes were identified as described above to provide an indication of the number and types of genes showing differential susceptibility to regulation by their corresponding peak motif in the two morphological leaf types. A statistical plot of genes displaying such significantly differential susceptibilities is presented in (Figure 12). Genes with differential susceptibilities for regulation by differentially available peak motifs were for subsequently subjected to GO (Figure 13) and KO enrichment analyses (Figure 14).

### 3.9 Enrichment analysis of exposed TF‐motifs in simple and lobed leaves

TF motif analysis for intergroup differential peaks assisted the identification of TFs involved in high and low variation in genomic openness between simple and lobed leaves. The MEME Suite was used as described above. Up‐regulated peak motif de novo prediction, using MEME software, from scratch to predict longer motif (8–15 bp) (Figure S12 A) down‐regulate the motif denovo prediction of peak. The following is the long motif (8–15 bp) predicted from scratch using MEME software (Figure S12 B), using Dreme software, and the longer motif predicted from scratch Short motif (3–8 bp) (Figure S12 C). The total number of motifs with an up‐regulated number of open chromatin sites is 6, whereas the number of down‐regulated sites is 24 (Figure S13).

## 4 DISCUSSION

The mulberry tree is a very important economic tree species, which is widely planted in the world. Its various organs have different economic or medicinal values, especially mulberry leaves, which are an important food source for silkworms (Ruth et al., 2019). In this study, we have utilized ATAC‐seq to analyze differences in chromatin accessibility between lobed and simple leaves of mulberry in order to identify regulatory mechanisms underlying the development of heteromorphic leaf shapes in *M. alba* individuals.

ATAC‐seq, which identifies open chromatin regions, identifies nucleosome bound and unbound sites enriched in regulatory regions including TFs. The bacterial transposase, Tn5, binds to the open regions of chromatin and cleaves it, whereas the helically wound portion is unaffected. Tn5 has been widely used in library construction for second‐generation sequencing and with the unique “tagging” function of the dimeric Tn5, it can cut double‐stranded DNA and ligate the resulting DNA ends to specific ligands. The cleaved and interrupted DNA is collected together for library construction and sequencing to characterize the remodeling of chromatin accessibility that occurs between biological conditions (Gontarz et al., 2020). Before ATAC‐Seq technology was developed, there were several commonly used methods to study open chromatin, such as DNase‐seq, MNase‐seq, and FAIRE‐seq.

DNase‐seq utilizes a restriction endonuclease (DNase I) to fragment the sample. In chromatin dense regions, DNA strands are well protected making it impossible for endonucleases to approach these regions so that only DNA in open regions are cut. Similarly, within the open regions of chromatin, DNA wound around nucleosomes is protected so that only DNA sequences between nucleosomes can be cut by DNase I (Zhong et al., 2016). MNase‐seq is similar to DNase‐seq in that it uses a restriction exonuclease to excises all unprotected regions, leaving only the DNA sequence wound on the nucleosome (Hu et al., 2017). Formaldehyde‐assisted isolation of regulatory elements sequencing (FAIRE‐seq) is a genome‐wide method for identifying chromatin accessibility and detecting DNA sequences associated with regulatory activity (Bianco et al., 2015). These techniques have obvious shortcomings, such as the very large number of cells required, the tedious experimental procedure, and the less coverage of data obtained by sequencing compared to ATAC‐Seq. In comparison, ATAC‐Seq starts with a low cell volume, takes less time and obtains a larger range of open regions. ATAC‐seq can capture the entire open area, but the sequencing results cannot match the results of DNase‐seq and MNase‐seq (Martins et al., 2018). In addition, the preparation of the library building is more tedious and the reagents are more expensive.

More TCP family transcription factor peaks are indicated in the results of this investigation. Numerous research on the regulation of leaf shape development by transcription factors of the TCP family has been reported. TCP family genes encode a set of plant‐specific transcription factors involved in multiple aspects of plant development (Barkoulas et al., 2007; Koyama et al., 2010; Lan & Qin, 2020).TCP affects leaf size and shape in many herbaceous plants. However, whether these functions are retained in woody plants remains unknown. Genome‐wide identification of TCP genes in *Populus* and *Populus trichocarpa* identified 33 and 36 genes encoding putative TCP proteins, respectively, and phylogenetic analysis of *Populus* TCP together with *Arabidopsis* TCP showed a biased expansion of the TCP gene family through segmental duplication. In addition, the results showed correlations between different expression patterns of several poplar TCP genes and leaf shape changes, suggesting their involvement in regulating leaf shape development (Ma et al., 2016). In this study, as shown in Figure 7, there were more TCP families, and most were enriched in the CK group, suggesting the possibility of TCP may be decreased in the HL group, resulting in the change of leaf shape (from whole leaves to lobed leaves). Also, the TCP family analyzed from the down‐regulated peaks in the HL group showed similar patterns (Figure S13B). This further supports the possibility that the expression of the TCP transcription factors family is reduced in cleaved leaves. The results showed that the TCP family is an important transcription factor that leads to different leaf shapes.

ATAC‐Seq technology itself will be used in new applications with the continuing development of other technologies, such as sciatica‐Seq which, combines single‐cell sequencing technology with it. Using ATAC‐Seq technology, we can solve a variety of problems, such as studying the pathogenesis of major diseases, finding differences in chromatin open regions of diseases; studying differences in binding regulation of TFs; identifying novel enhancers in development; and performing nucleosome localization (Ji et al., 2020; Martins et al., 2018; Satpathy et al., 2018). In addition, this technique can be used in combination with other techniques, such as ChIP‐seq, which can be used to validate potential binding regions of TFs predicted by ATAC‐Seq (Lhoumaud et al., 2019; Newell et al., 2021). Alternatively, it can be combined with transcriptome sequencing to combine differentially expressed genes with Peak results to see the corresponding expression of transcripts and to study the upstream regulatory sequences, all of which are hot topics of current research (Ranzoni et al., 2021; Xu et al., 2022).

## 5 CONCLUSION

In this study, we used ATAC‐seq to mine the TFs that lead to leaf shape differences, detected and counted single samples and intra‐group peaks respectively by bioinformatics analysis and performed TF motif analysis to provide insight the TFs related to heteromorphic leaf occurrence. This study has laid the foundation for an in‐depth study to understand the mechanisms underlying leaf shape changes in mulberry.

## AUTHOR CONTRIBUTIONS

Weiguo Zhao, Xuzhu Gao, and Dayong Xu conceived this study. Lei Wang, Yuming Feng, and Jiangying Wang performed the experiments. Lei Wang and Michael Ackah wrote the manuscript. Xin Jin and Qiaonan Zhang revised the original manuscript. All authors read and approved the final version of the manuscript.

## CONFLICT OF INTEREST

The Authors did not report any conflict of interest.

## Supporting information


**Table S1** Statistical results of mitochondria and chloroplasts for each sample comparison
Table S2 Statistics of the number of peaks in each group
Table S3 Distribution statistics of peaks on functional elements of genes
Table S4 Sample TF motif number statistics
Table S5 qRT‐PCR primer sequences
Table S6 Number of grouped TF motif statistics. Subgroup CK‐vs‐HL.CK motif enrichment statistics
**Figure S1**: Base distribution before and after filtering, A‐D based distribution before filtering, E‐H base distribution after filtering
**Figure S2**: Cumulative analysis of genome sequencing depth. A. CK‐1 sequencing depth, B. CK‐2 sequencing depth, C. HL‐1 sequencing depth, D. HL‐2 sequencing depth
**Figure S3**: Distribution of reads on chromosomes. A. Distribution of CK‐1 reads on chromosomes, B. Distribution of HL‐1 reads on chromosomes, C. Distribution of CK‐2 reads on chromosomes, D. Distribution of HL‐2 reads on chromosomes
**Figure S4**: HL GO enrichment analysis. A. GO enrichment circle graph, B. GO enrichment bubble graph, C. GO enrichment bar graph, D. GO enrichment classification bar graph
**Figure S5**: CK GO enrichment analysis. A. GO enrichment circle graph, B. GO enrichment bubble graph, C. GO enrichment bar graph, D. GO enrichment classification bar graph
**Figure S6**: HL KO enrichment analysis. A. KO enrichment circle graph, B. KO enrichment bubble graph, C. KO enrichment bar graph
**Figure S7**: CK KO enrichment analysis. A. KO enrichment circles, B. KO enrichment bubbles, C. KO enrichment bars
**Figure S8**: GO enrichment analysis. A. GO enrichment circle graph, B. GO enrichment classification bar graph, C. GO enrichment bubble graph, D. GO enrichment bar graph
**Figure S9**: KO enrichment analysis. A. KO enrichment circle, B. KO enrichment bubble, C. KO enrichment bar graph
**Figure S10**: A/B MEME significant motif sequence, C/D. Dreme significant motif sequence
**Figure S11**: Grouped motif‐enriched bubble plots
**Figure S12**: TF‐motif denovo prediction. A. Up‐regulate peeks motif denovo prediction, B. Down‐regulate peak's motif denovo prediction, C. Down‐regulate peaks motif denovo prediction (3‐8 bp).
**Figure S13**: Comparison group motif enrichment bubble plot.
