# Supplementary material for: ATAC‐seq exposes differences in chromatin accessibility leading to distinct leaf shapes in mulberry
Source: Plant Direct. 2022 Dec 15;6(12):e464. doi: 10.1002/pld3.464 (PMC9755926; doi:10.1002/pld3.464)
Supplement: Supplementary file 1 — Table S1 Statistical results of mitochondria and chloroplasts for each sample comparison Table S2 Statistics of the number of peaks in each group Table S3 Distribution statistics of peaks on functional elements of genes Table S4 Sample TF motif number statistics Table S5 qRT‐PCR primer sequences Table S6 Number of grouped TF motif statistics. Subgroup CK‐vs‐HL.CK motif enrichment statistics Figure S1: Base distribution before and after filtering, A‐D based distribution before filtering, E‐H base distribution after filtering Figure S2: Cumulative analysis of genome sequencing depth. A. CK‐1 sequencing depth, B. CK‐2 sequencing depth, C. HL‐1 sequencing depth, D. HL‐2 sequencing depth Figure S3: Distribution of reads on chromosomes. A. Distribution of CK‐1 reads on chromosomes, B. Distribution of HL‐1 reads on chromosomes, C. Distribution of CK‐2 reads on chromosomes, D. Distribution of HL‐2 reads on chromosomes Figure S4: HL GO enrichment analysis. A. GO enrichment circle graph, B. GO enrichment bubble graph, C. GO enrichment bar graph, D. GO enrichment classification bar graph Figure S5: CK GO enrichment analysis. A. GO enrichment circle graph, B. GO enrichment bubble graph, C. GO enrichment bar graph, D. GO enrichment classification bar graph Figure S6: HL KO enrichment analysis. A. KO enrichment circle graph, B. KO enrichment bubble graph, C. KO enrichment bar graph Figure S7: CK KO enrichment analysis. A. KO enrichment circles, B. KO enrichment bubbles, C. KO enrichment bars Figure S8: GO enrichment analysis. A. GO enrichment circle graph, B. GO enrichment classification bar graph, C. GO enrichment bubble graph, D. GO enrichment bar graph Figure S9: KO enrichment analysis. A. KO enrichment circle, B. KO enrichment bubble, C. KO enrichment bar graph Figure S10: A/B MEME significant motif sequence, C/D. Dreme significant motif sequence Figure S11: Grouped motif‐enriched bubble plots Figure S12: TF‐motif denovo prediction. A. Up‐regulate peeks motif denovo predictio [file PLD3-6-e464-s001.docx]

**Supplementary materials**

Supplementary Table S1 Statistical results of mitochondria and chloroplasts for each sample comparison

| **Sample** | **Total_Reads** | **Mapped_Reads** | **Unmapped_Reads** | **Mitochondria_Reads** | **Chloroplast_Reads** |
| --- | --- | --- | --- | --- | --- |
| CK-1 | 173083490 | 0(0.00%) | 173083490(100.00%) | 0(0.00%) | 0(0.00%) |
| CK-2 | 143046708 | 0(0.00%) | 143046708(100.00%) | 0(0.00%) | 0(0.00%) |
| HL-1 | 168445512 | 0(0.00%) | 168445512(100.00%) | 0(0.00%) | 0(0.00%) |
| HL-2 | 155636996 | 0(0.00%) | 155636996(100.00%) | 0(0.00%) | 0(0.00%) |

Supplementary Table S2 Statistics of the number of peaks in each group

| **Sample** | **Peak_Number** | **Total_Length** | **Average_Length** | **Genome_Ratio** |
| --- | --- | --- | --- | --- |
| CK | 26471 | 13677960 | 516 | 4.62% |
| HL | 21278 | 10387908 | 488 | 3.51% |

Supplementary Table S3 Distribution statistics of peaks on functional elements of genes

| **Sample** | **peakCount** | **promoter** | **5'UTR** | **3'UTR** | **1st_exon** | **other_exon** | 1st_intron | **ohter_intron** | **downstream** | **distal_intergenic** |
| --- | --- | --- | --- | --- | --- | --- | --- | --- | --- | --- |
| CK | 26471 | 7266-27.45% | 1992-7.53% | 705-2.66% | 1620-6.12% | 2955-11.16% | 676-2.55% | 1379-5.21% | 1798-6.79% | 8080-30.52% |
| HL | 21278 | 5811-27.31% | 1747-8.21% | 441-2.07% | 1766-8.30% | 2453-11.53% | 490-2.30% | 926-4.35% | 1259-5.92% | 6385-30.01% |

Supplementary Table S4 Sample TF motif number statistics

| SampleId | motifNumber |
| --- | --- |
| CK | 65 |
| HL | 52 |

Supplementary Table S5 qRT-PCR primer sequences

| **Gene** | **Primer** | **Sequence (5’-3’)** |
| --- | --- | --- |
| *β*- actin | forward primer | CCGTTCTCTCCCTTTACGCC |
|  | reverse primer | AGACGGAGAATAGCATGGGGA |
| ABF2 | forward primer | GCTCTTTCCCAAACC |
|  | reverse primer | GTTCCCGCACTCATA |
| ABI5 | forward primer | AACCACCCGTTCTCG |
|  | reverse primer | GCGGTCCAAATGCTG |
| ARALYDRAFT_484466 | forward primer | GGCTTTGACTTCCCTC |
|  | reverse primer | ATATGGCCGTCTTGC |
| ARALYDRAFT_495258 | forward primer | GCCCATCAAGACAGCA |
|  | reverse primer | GATTAGCGACGAAGGAA |
| At1g72010 | forward primer | AACACCTCCAACACCA |
|  | reverse primer | AAAGGGACCACAAACTG |
| AT4G18890 | forward primer | TTTCAGCCTTGTCTCG |
|  | reverse primer | CATCCGCCGTGTTAT |
| AT5G08330 | forward primer | CGCCTCCACGACTTCCT |
|  | reverse primer | TGCCTCCAACCACTGAAAC |
| bHLH31 | forward primer | GTTTGGCTCCGTTTG |
|  | reverse primer | CATCTTCCACCACATCA |
| Glyma19g26560.1 | forward primer | ATCCCAAGGGCTCAA |
|  | reverse primer | TCCGCAGGTTTGTTT |
| HY5 | forward primer | ACTTGAGTGACCTGGAA |
|  | reverse primer | AACCACCACTACCTCC |
| MYC3 | forward primer | GATTTATGTCGGCTTTG |
|  | reverse primer | TTCACCGTCGCTTGT |
| OJ1058_F05.8 | forward primer | TGACGCTGGAGGACTT |
|  | reverse primer | CTCGGTCAACTGAGGC |
| OsI_08196 | forward primer | CGCCTCCACGACTTCCT |
|  | reverse primer | TGCCTCCAACCACTGAAAC |
| PIF1 | forward primer | GACGACCCTTCCTTG |
|  | reverse primer | CGAGTTGATGGCTGTG |
| PIF4 | forward primer | GGATGGGAAGTGGTA |
|  | reverse primer | GGATAGAAGGCAAGG |
| PIF7 | forward primer | ACGCCAGCCACCATA |
|  | reverse primer | TCCGCTTTCTCATCACTAA |
| TCP19 | forward primer | ATCGCCGAAGTCCTG |
|  | reverse primer | ACCAACCCTTGTGCC |
| TCP20 | forward primer | GGCTTTGACTTCCCTC |
|  | reverse primer | ATATGGCCGTCTTGC |
| TCP23 | forward primer | AACACCTCCAACACCA |
|  | reverse primer | AAAGGGACCACAAACTG |
| TCP7 | forward primer | CGCCTCCACGACTTCCT |
|  | reverse primer | TGCCTCCAACCACTGAAAC |

Supplementary Table S6 Number of grouped TF motif statistics

| Sample Id | Motif Number |
| --- | --- |
| CK-vs-HL_CK | 35 |
| CK-vs-HL_HL | 77 |

Supplementary Table S6 Subgroup CK-vs-HL.CK motif enrichment statistics

| **motif_ID** | **motif_alt_ID** | **consensus** | **adj_p-value** | **E-value** | **TP** | **%TP** | **FP** | **%FP** |
| --- | --- | --- | --- | --- | --- | --- | --- | --- |
| MA0120.1 | id1 | TTKYYYYTHBCG | 2.78e-10 | 1.36e-7 | 223 | 55.75 | 422 | 35.17 |
| MA1192.1 | At5g58900 | WDWRGATAAGRTTWD | 4.35e-10 | 2.13e-7 | 54 | 13.50 | 39 | 3.25 |
| MA0989.1 | PHYPADRAFT_153324 | NNWAAAGBN | 1.43e-9 | 6.99e-7 | 113 | 28.25 | 150 | 12.50 |
| MA1022.1 | PHYPADRAFT_38837 | NNWAAAGBNN | 3.84e-9 | 1.88e-6 | 142 | 35.50 | 220 | 18.33 |
| MA0940.1 | AP1 | MYAAAAAWRGAAA | 1.00e-8 | 4.90e-6 | 200 | 50.00 | 375 | 31.25 |
| MA1186.1 | At1g49010 | HWWAWYCTTATCYWH | 1.20e-8 | 5.87e-6 | 62 | 15.50 | 58 | 4.83 |
| MA0956.1 | BEE2 | NNCACGTGNN | 2.07e-8 | 1.01e-5 | 120 | 30.00 | 178 | 14.83 |
| MA0045.1 | HMG-I/Y | VWAVAAAHRVMRAMAY | 2.11e-8 | 1.03e-5 | 209 | 52.25 | 403 | 33.58 |
| MA1399.1 | At5g08520 | DWWDWRGATAAGR | 2.27e-8 | 1.11e-5 | 50 | 12.50 | 40 | 3.33 |
| MA0981.1 | DOF1.8 | NNWAAAGBNN | 1.08e-7 | 5.28e-5 | 154 | 38.50 | 263 | 21.92 |

**Supplementary Figure &Figure legends**

**
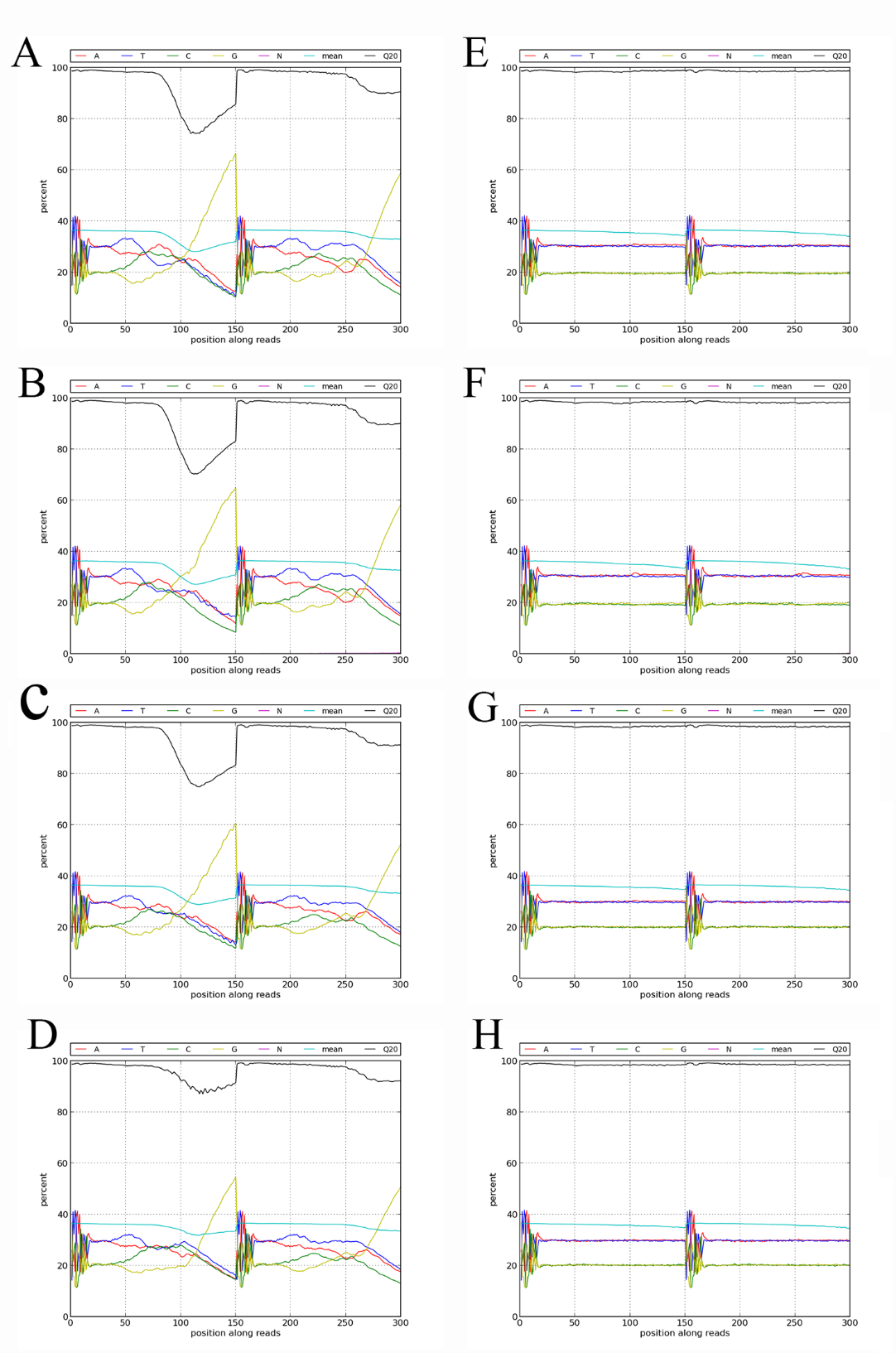
**

**Supplementary Figure S1**: Base distribution before and after filtering, A-D based distribution before filtering, E-H base distribution after filtering


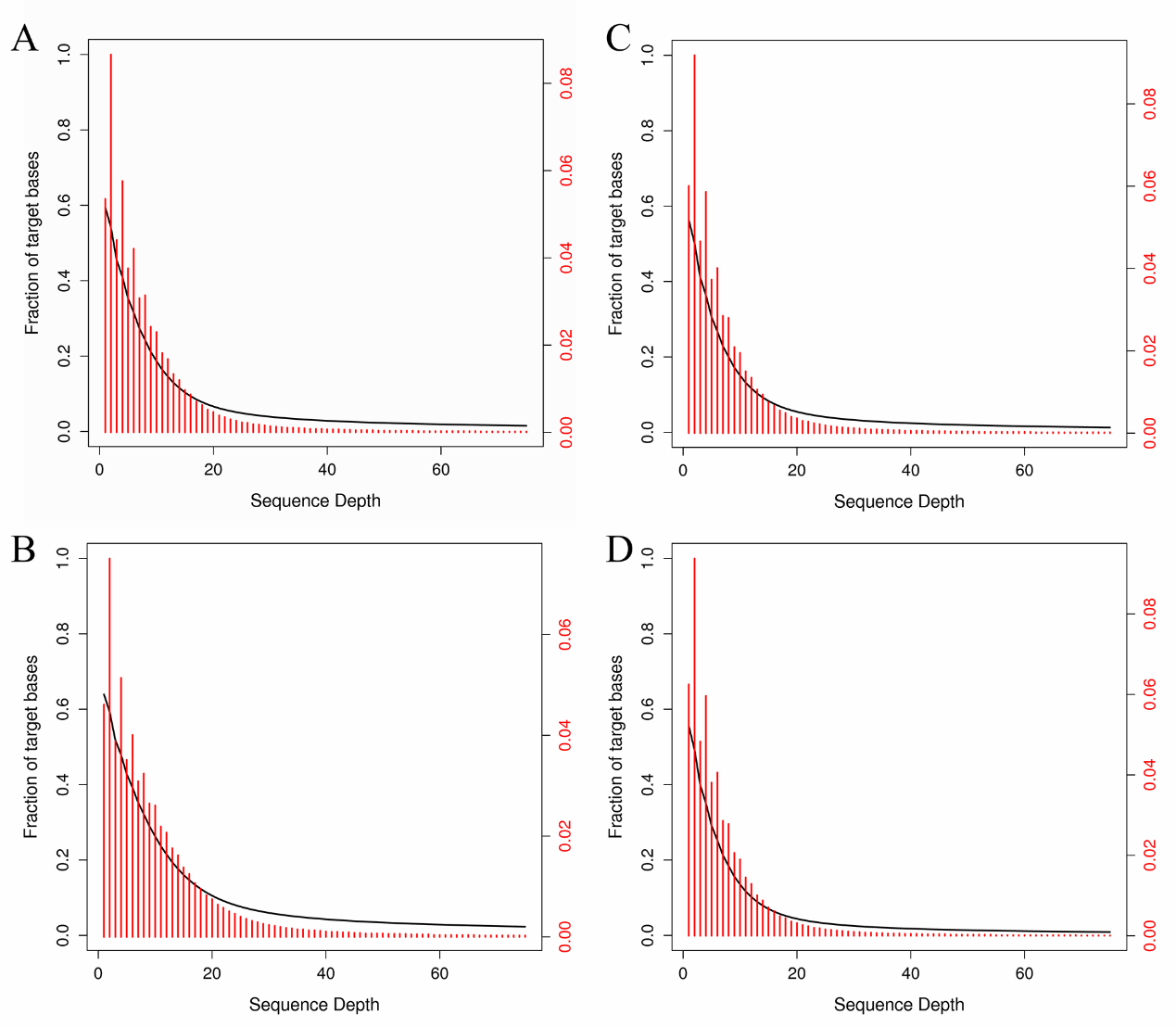


**Supplementary Figure S2**: Cumulative analysis of genome sequencing depth. A. CK-1 sequencing depth, B. CK-2 sequencing depth, C. HL-1 sequencing depth, D. HL-2 sequencing depth


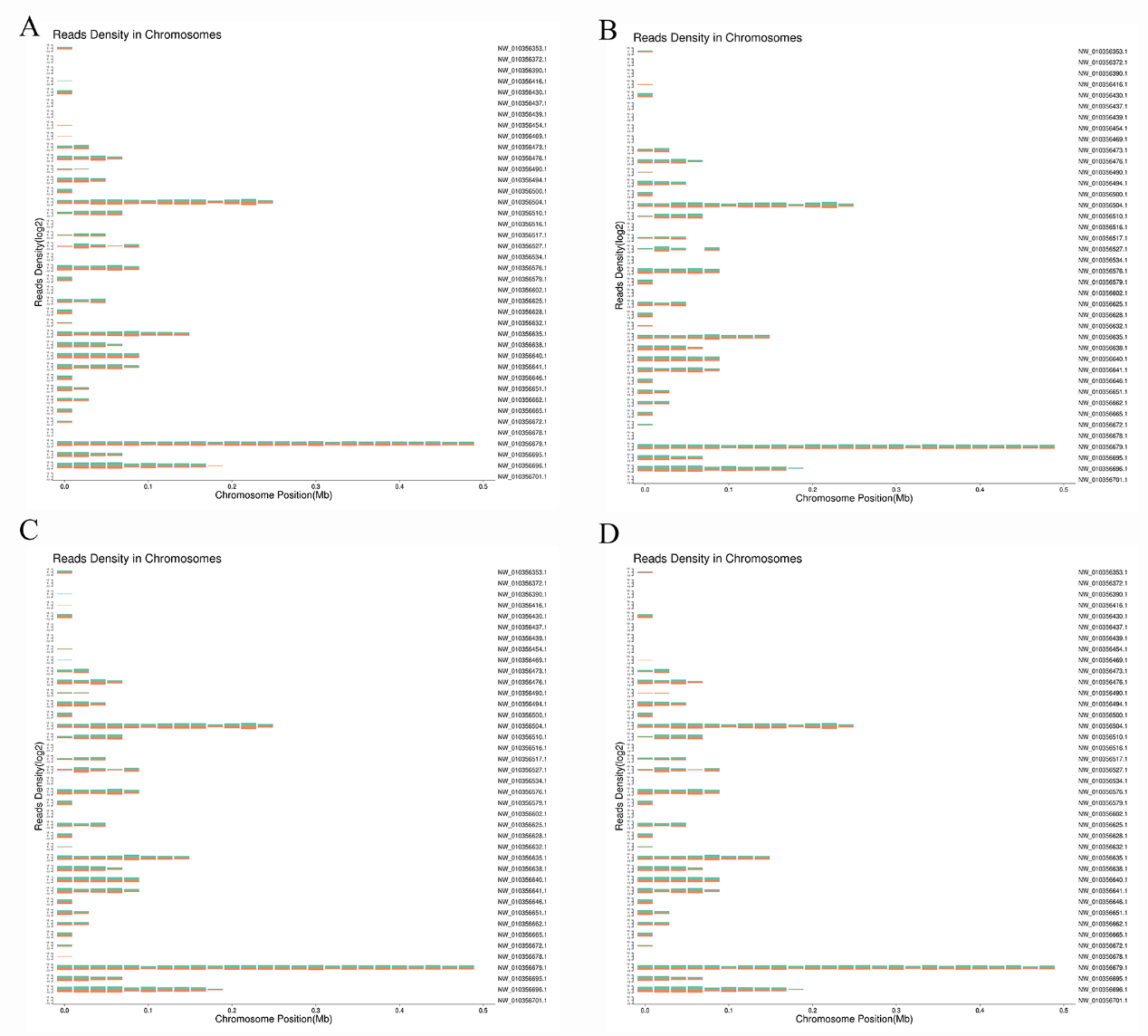


**Supplementary Figure S3**: Distribution of reads on chromosomes. A. Distribution of CK-1 reads on chromosomes, B. Distribution of HL-1 reads on chromosomes, C. Distribution of CK-2 reads on chromosomes, D. Distribution of HL-2 reads on chromosomes


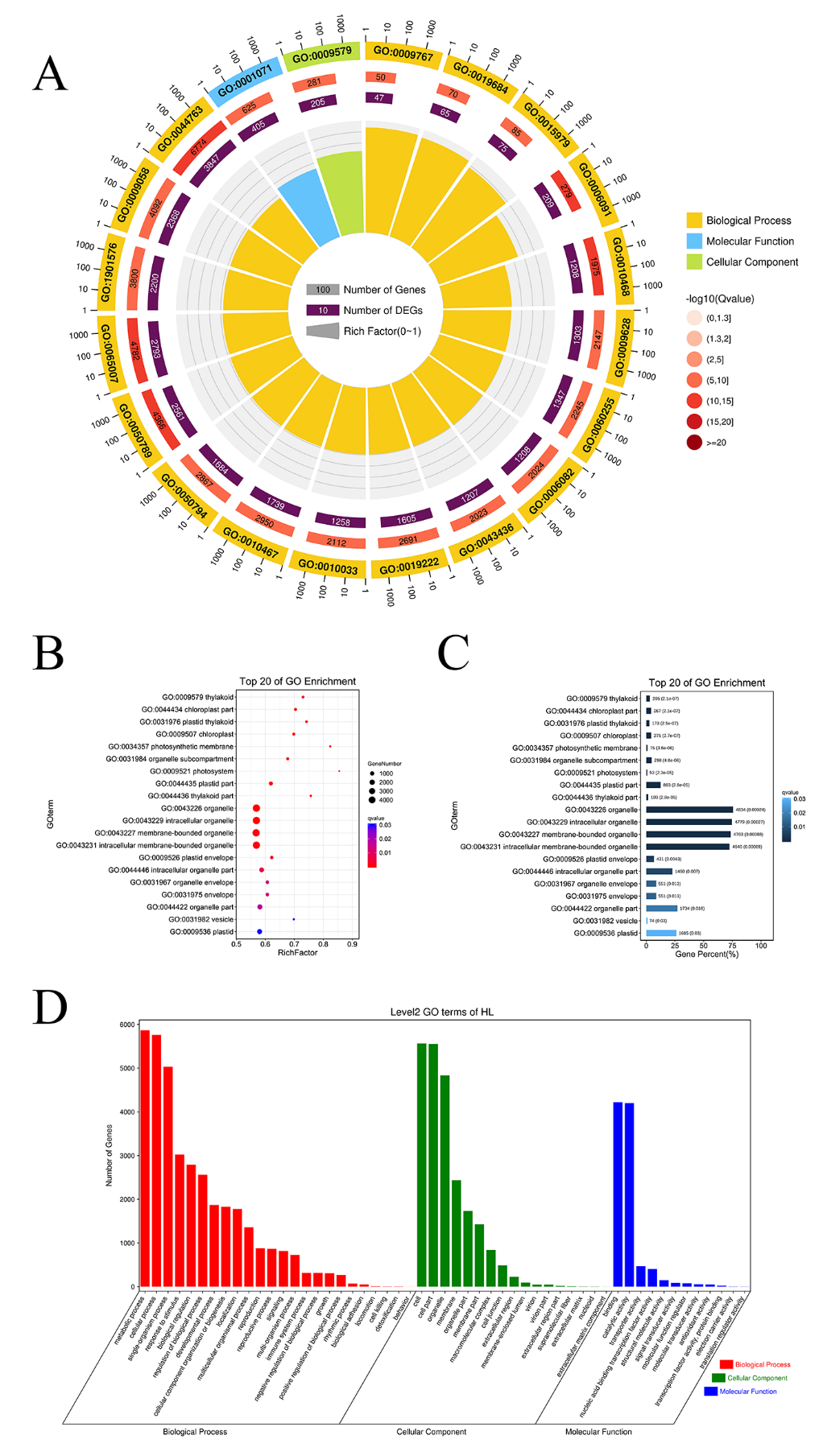


**Supplementary Figure S4**: HL GO enrichment analysis. A. GO enrichment circle graph, B. GO enrichment bubble graph, C. GO enrichment bar graph, D. GO enrichment classification bar graph


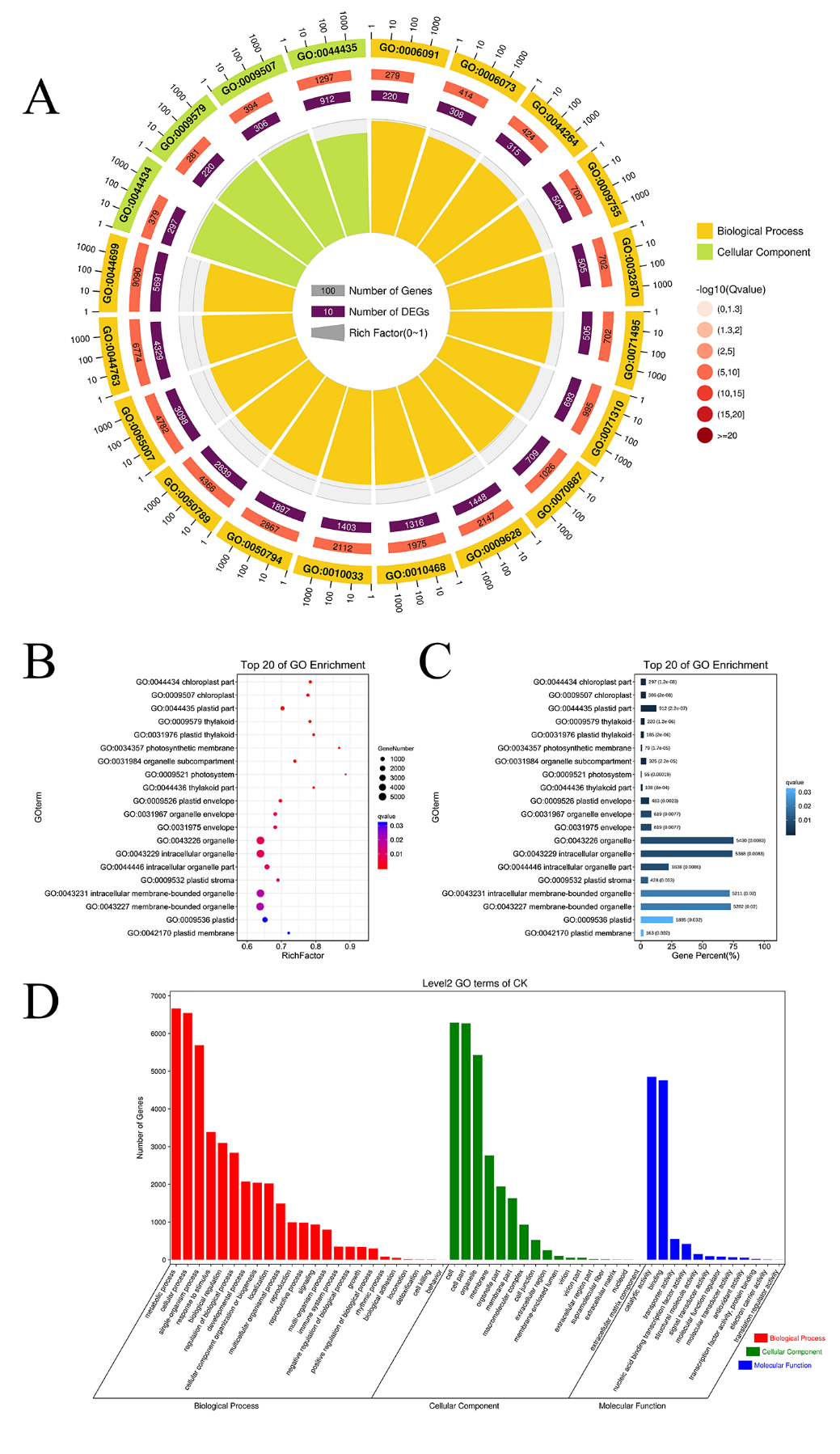


**Supplementary Figure S5**: CK GO enrichment analysis. A. GO enrichment circle graph, B. GO enrichment bubble graph, C. GO enrichment bar graph, D. GO enrichment classification bar graph


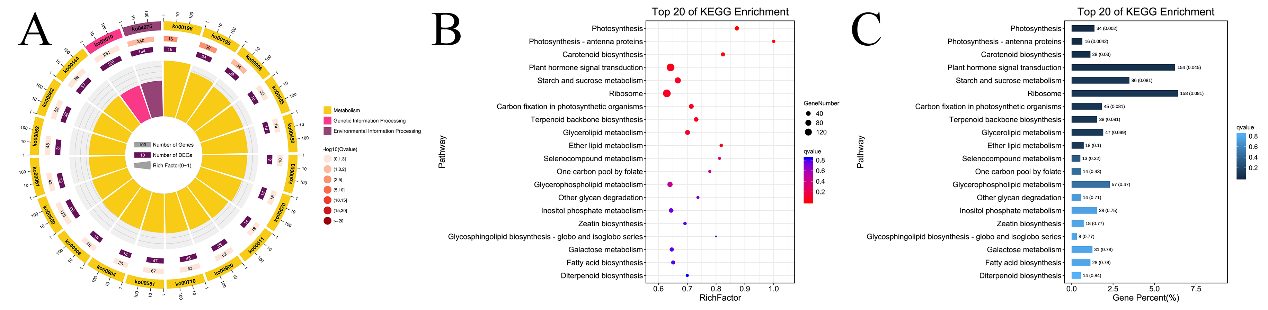


**Supplementary Figure S6**: HL KO enrichment analysis. A. KO enrichment circle graph, B. KO enrichment bubble graph, C. KO enrichment bar graph


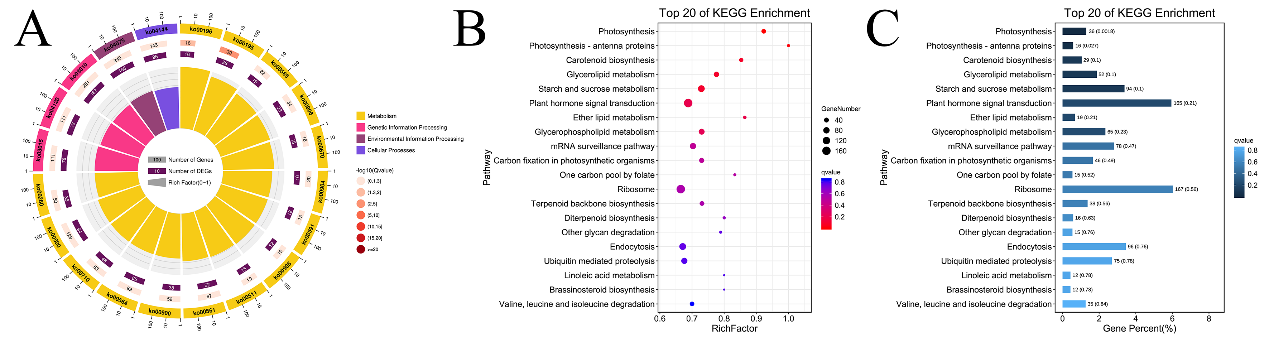


**Supplementary Figure S7**: CK KO enrichment analysis. A. KO enrichment circles, B. KO enrichment bubbles, C. KO enrichment bars


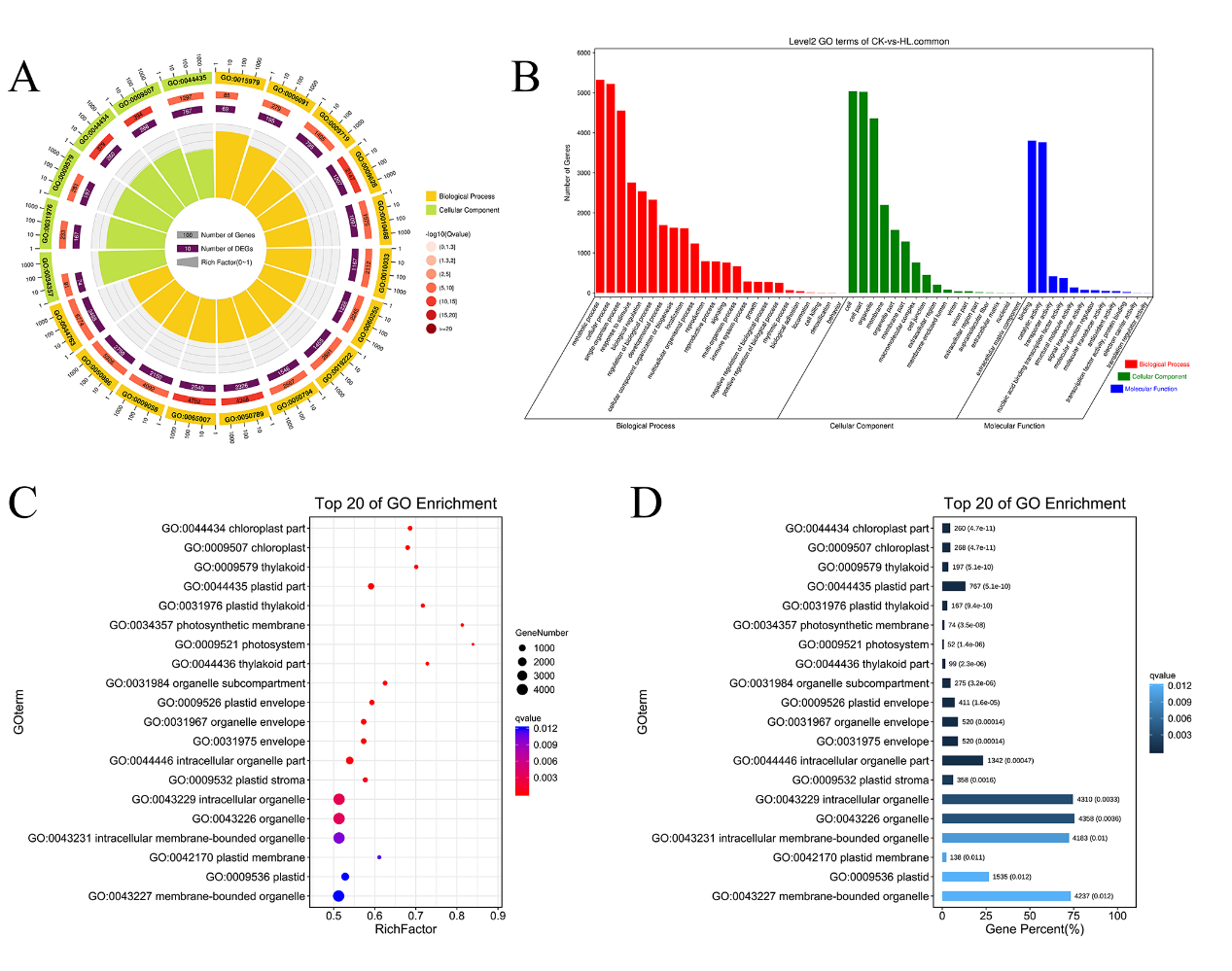


**Supplementary Figure S8**: GO enrichment analysis. A. GO enrichment circle graph, B. GO enrichment classification bar graph, C. GO enrichment bubble graph, D. GO enrichment bar graph


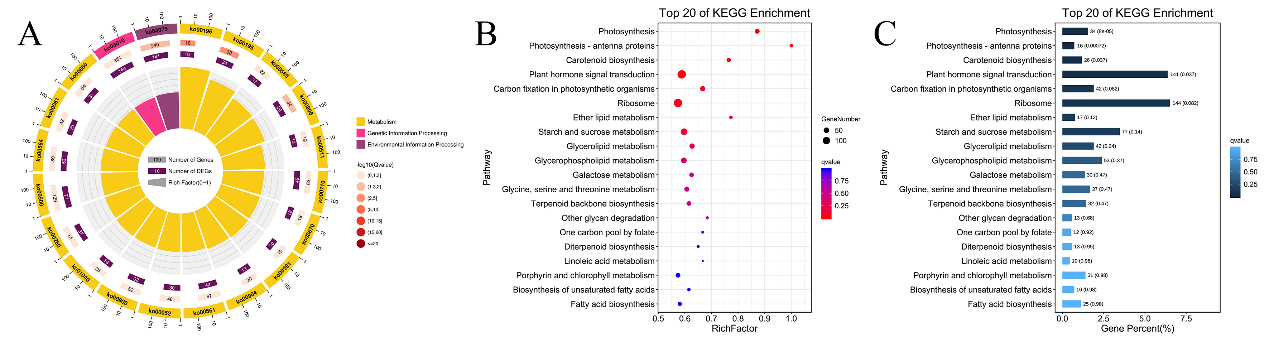


**Supplementary Figure S9**: KO enrichment analysis. A. KO enrichment circle, B. KO enrichment bubble, C. KO enrichment bar graph


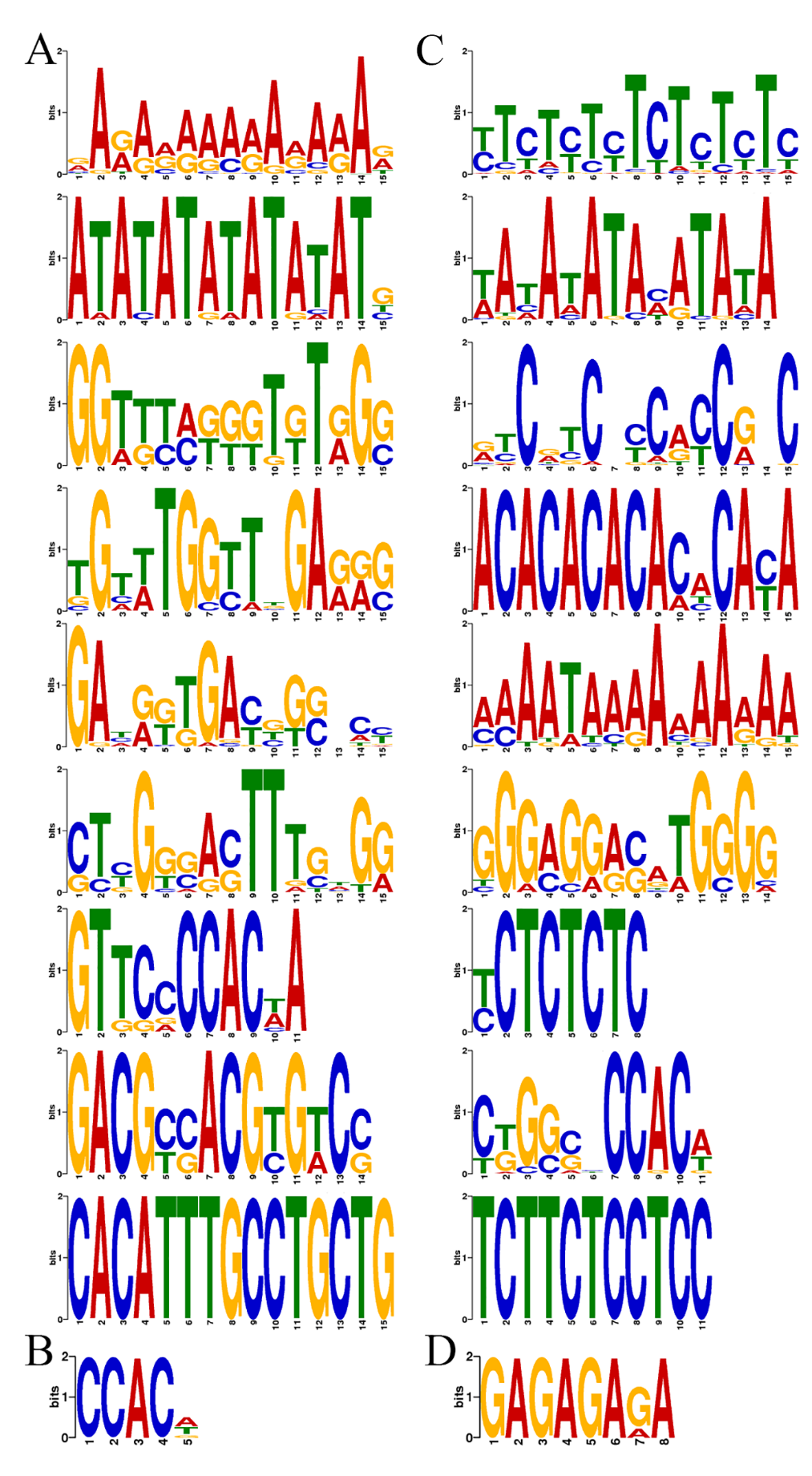


**Supplementary Figure S10**: A/B MEME significant motif sequence, C/D. Dreme significant motif sequence


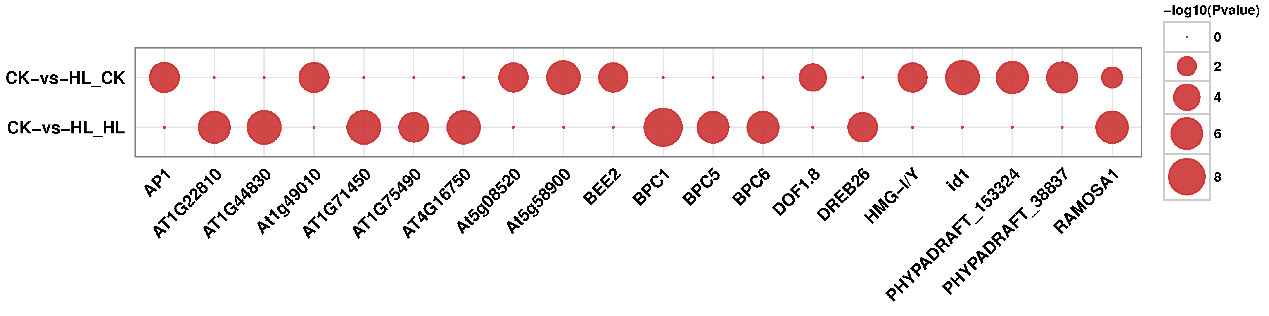


**Supplementary Figure S11**: Grouped motif-enriched bubble plots


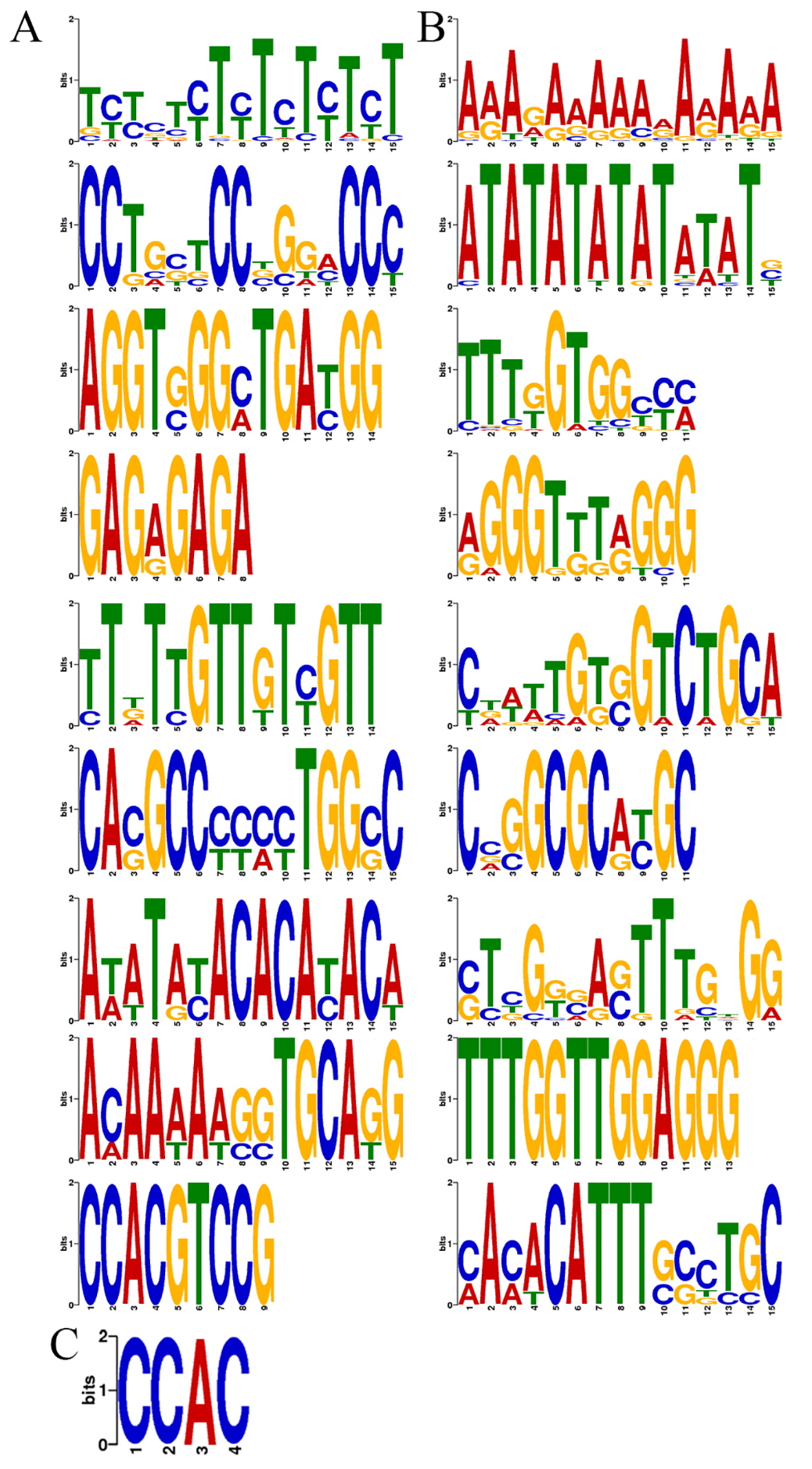


**Supplementary Figure S12**: TF-motif denovo prediction. A. Up-regulate peeks motif denovo prediction, B. Down-regulate peak's motif denovo prediction, C. Down-regulate peaks motif denovo prediction (3-8bp).


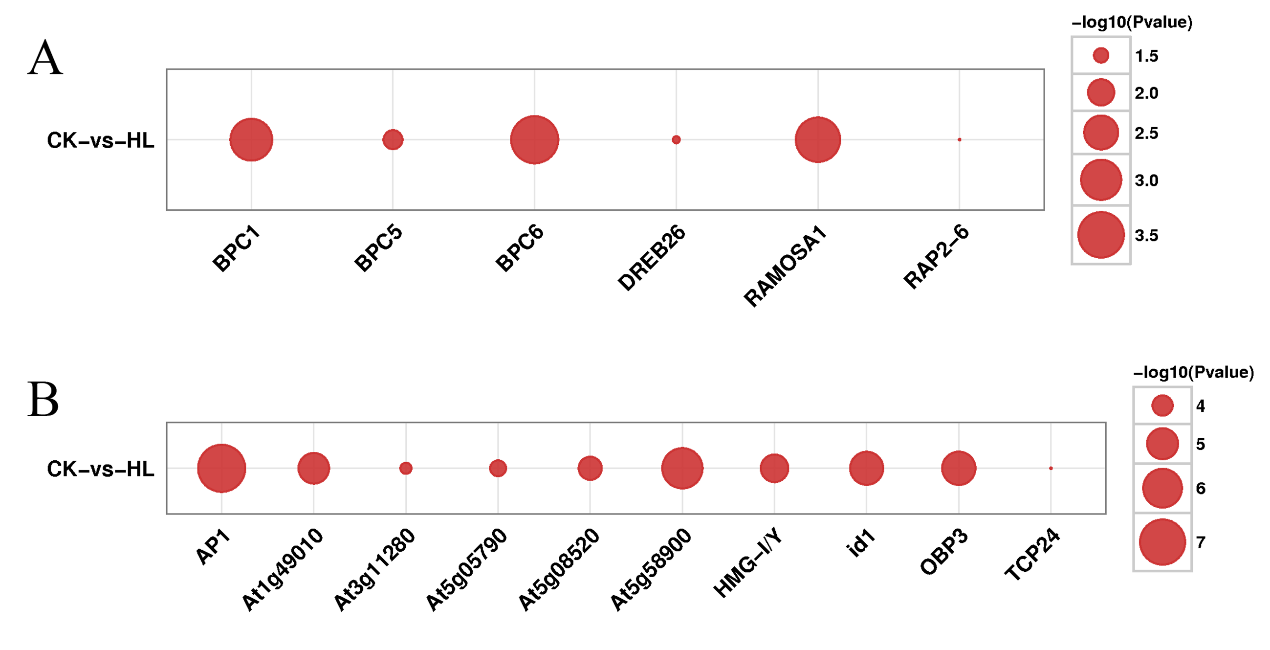


**Supplementary Figure S13**: Comparison group motif enrichment bubble plot.
